# Downregulation of low-density lipoprotein receptor mRNA in lymphatic endothelial cells impairs lymphatic function through changes in intracellular lipids

**DOI:** 10.7150/thno.58780

**Published:** 2022-01-01

**Authors:** Laurent Vachon, Ali Smaani, Nolwenn Tessier, Gabriel Jean, Annie Demers, Andreea Milasan, Nadine Ardo, Stéphanie Jarry, Louis Villeneuve, Azadeh Alikashani, Vincent Finherty, Matthieu Ruiz, Mary G. Sorci-Thomas, Gaétan Mayer, Catherine Martel

**Affiliations:** 1 Department of Medicine, Faculty of Medicine, Université de Montréal, Montreal, Quebec, Canada.; 2 Montreal Heart Institute, Research Center, Montreal, Quebec, Canada.; 3 Department of Nutrition, Faculty of Medicine, Université de Montréal, Montreal, Quebec, Canada.; 4 Montreal Heart Institute, Metabolomics platform, Montreal, Quebec, Canada.; 5 Department of Medicine, Medical College of Wisconsin, Milwaukee, Wisconsin, USA.; 6 Faculty of Pharmacy, Université de Montréal, Montreal, QC, Canada.

**Keywords:** Atherosclerosis, Lymphatic System, Lymphatic dysfunction, LDLR, PCSK9

## Abstract

**Rationale:** Impairment in lymphatic transport is associated with the onset and progression of atherosclerosis in animal models. The downregulation of low-density-lipoprotein receptor (LDLR) expression, rather than increased circulating cholesterol level *per se*, is involved in early atherosclerosis-related lymphatic dysfunction. Enhancing lymphatic function in *Ldlr^-/-^* mice with a mutant form of VEGF-C (VEGF-C 152s), a selective VEGFR-3 agonist, successfully delayed atherosclerotic plaque onset when mice were subsequently fed a high-fat diet. However, the specific mechanisms by which LDLR protects against lymphatic function impairment is unknown.

**Methods and results:** We have thus injected wild-type and *Pcsk9^-/-^* mice with an adeno-associated virus type 1 expressing a shRNA for silencing *Ldlr in vivo*. We herein report that lymphatic contractility is reduced upon *Ldlr* dowregulation in wild-type mice only. Our *in vitro* experiments reveal that a decrease in *LDLR* expression at the mRNA level reduces the chromosome duplication phase and the protein expression of VEGFR-3, a membrane-bound key lymphatic marker. Furthermore, it also significantly reduced the levels of 18 lipid subclasses, including key constituents of lipid rafts as well as the transcription of several genes involved in cholesterol biosynthesis and cellular and metabolic processes. Exogenous PCSK9 only reduces lymphatic endothelial-LDLR at the protein level and does not affect lymphatic endothelial cell integrity. This puts forward that PCSK9 may act upon lymphatic muscle cells to mediate its effect on lymphatic contraction capacity *in vivo*.

**Conclusion:** Our results suggest that treatments that specifically palliate the down regulation of *LDLR* mRNA in lymphatic endothelial cells preserve the integrity of the lymphatic endothelium and sustain lymphatic function, a prerequisite player in atherosclerosis.

## Introduction

Four decades ago, Lemole suggested that intimal arterial thickness was associated with lymphatic vessel blockage 1. He proposed that enhanced stagnation of the interstitial fluid in the arterial wall could be due to lymphostasis 1, 2. Since then, in addition to the well-described classical roles of the lymphatic network 3, 4, studies analyzing the morphology of lymphatic vessels in the artery wall shed light on their associations with atherosclerosis 5-8. Subsequently, functional studies have then proven that the absence of functional adventitial lymphatics is associated to an exacerbated atherosclerotic plaque accumulation in mice 9, 10. It is now well accepted that an efficient lymphatic drainage is crucial for atherosclerosis regression 9, 11, 12. The first studies on the topic were suggesting that hypercholesterolemia was at the corner stone between cardiovascular disease and lymphatic function 10, 13, 14. Recently, the role of the lymphatics has been refined throughout the development of the atherosclerosis process, placing front stage the potential role of an early lymphatic dysfunction before the onset of the disease 15, 16. Our team has reported that lymphatic function impairment occurs even before the onset of atherosclerosis in *Ldlr^-/-^*; hapoB100*^+/+^* mice 16. The absence of low-density lipoprotein receptor (LDLR), but not the increase in circulating cholesterol levels, is potentially instigative of this early atherosclerosis-related collecting lymphatic vessel dysfunction 16. We have subsequently found that enhancing collecting lymphatic vessels with a mutant form of the vascular endothelial growth factor-C (namely VEGF-C 152S) early on in the atherosclerotic process protects from subsequent excessive plaque formation in *Ldlr^-/-^* mice 15. On the other hand, our team has also demonstrated for the first time that a systemic knockout in proprotein convertase subtilisin kexin type 9 (PCSK9) improves lymphatic transport in conjunction with increasing LDLR expression in lymphatic endothelial cells (LEC) in six-month-old mice compared to wild-type mice 16. As PCSK9 deletion did not affect lymphangiogenesis *per se*, these results suggest that collecting lymphatic vessels are primarily involved.

PCSK9 is capable of binding LDLR, leading to its internalization and degradation in lysosomes 17. The degradation of this complex is regulated by PCSK9 and LDLR levels, but also by a variety of other proteins 18-20. PCSK9 also has pleiotropic effects including in cardiovascular metabolism. It has been reported to promote the internalization and degradation of other receptors and members of the LDLR superfamily 21-25. Albeit PCSK9 has been extensively studied in plasma 26, 27, it is only recently that its accumulation in systemic lymph has been reported 16. Whether PCSK9 is produced by LEC or if it affects lymphatic function via LDLR binding or through other pleiotropic mechanisms is not known yet. Using an *in vivo* model of induced endothelial deletion of *Ldlr*, we herein report that lymphatic contractility is reduced upon *Ldlr* dowregulation in wild-type male mice, but not in *Pcsk9^-/-^* mice. *LDLR* expression had to be decreased at the mRNA level to observe a reduction in the chromosome duplication phase and in the protein expression of VEGFR-3, a membrane-bound key lymphatic marker. Exogenous PCSK9 only reduces LEC-LDLR at the protein level and does not affect lymphatic endothelial cell integrity. This put forward that PCSK9 may act upon lymphatic muscle cells to mediate its effect on lymphatic contraction capacity *in vivo*. Lipidomic and transcriptomic analysis further revealed that the levels of crucial constituents of lipid rafts and the transcription of several genes involved in cholesterol biosynthesis and cellular and metabolic processes were also reduced upon *LDLR* mRNA downregulation in LEC. Globally, our study suggests that treatments that specifically palliate the down regulation of *LDLR* mRNA in lymphatic endothelial cells might preserve the integrity of the lymphatic endothelium and sustain lymphatic function, a prerequisite player in atherosclerosis.

## Materials and methods

### Adeno-associated virus (AAV) vector constructs

Single-stranded cDNA (sscDNA) containing different short hairpin RNA (shRNA) flanked by HindIII and BamHI sites and two linkers were purchased from IDT and amplified by PCR using forward and reverse linker primers (Table 1). Double-stranded amplicons were digested with the two restriction sites, gel-purified and ligated in pU6-ITR, followed by transformation in Stbl3 bacteria. All used clones were confirmed by sequencing and purified by Maxiprep (Qiagen, Catalog No. D4202).

### AAV type 1 (AAV1) production and purification

Human embryonic kidney 293T (HEK 293T) cells were seeded in sixteen 100 mm plates in DMEM without sodium pyruvate (Wisent cat. 319-015) supplemented with 10% heat-inactivated fetal bovine serum (FBS; Wisent cat. 080-150). Once they had reached 70-80% confluence, cells were transfected with 8.6 µg/plate of the packaging plasmid pXYZ and 2.9 µg/plate of the specific pU6-ITR-sh plasmid (pU6-ITR-AAV1-sh-mLdlr or pU6-ITR-AAV1-sh-scramble) using the polyethylenimine method (46 µL/plate, PEI; Polysciences, cat. 23966). Cells were harvested 72 h post-transfection with a cell scraper, pelleted, resuspended in Tris lysis buffer (150 mM NaCl, 50 mM Tris pH 8.5) and subjected to three freeze/thaw cycles. Cell lysates were treated with 1 mM of MgCl_2_ and 250 units of benzonase (Santa Cruz cat. sc-202391) for 45 min at 37 °C and then centrifuged 20 min at 6000 g to remove cell debris. The lysate was purified by iodixanol gradient. Briefly, 60% iodixanol (OptiPrep; Sigma D1556) was diluted to 40% and 25% in 5X PBS-MK (5X PBS, 5 mM MgCl_2_, 12.5 mM KCl). Then, 2.5 µL of 0.5% phenol red (Bioshop PHE600.5/5g) was added to the 25 and 60% iodixanol phase. One milliliter of 60, 40, and 25% iodixanol solutions was successively added in a 5-mL Beckman ultracentrifuge tube (catalog No# 326819), and 1.5 to 2 mL of the processed crude lysate was gently overlaid onto the gradients and centrifuged in a 55Ti rotor for 3 h at 33000 rpm at 4 °C. After centrifugation, the tubes were punctured at the 60% and 40% interphase (bottom and middle layers) using a 18-gauge needle, to collect ~1-mL/tube of the fraction containing the virus. A pool of collected iodixanol containing virus was cleaned with PBS-0.001% Tween and concentrated with an Amicon Ultra-15 centrifugal filter unit (MWCO 100kDa; Merck Millipore cat. UFC910024). The virus was then titrated by quantitative PCR using AAV ITR primers (Table 1) and a standard curve using the corresponding pU6-ITR-sh plasmid in serial dilutions. Finally, viral batches were aliquoted and stored at -80 °C.

### Experimental mouse model

C57BL/6 wild-type and *Pcsk*9 knockout (*Pcsk*9^-/-^) mice were obtained from the Jackson Laboratory. The *Pcsk*9^-/-^ mice were backcrossed on a C57BL/6 background for at least 10 generations at our facility. Animals were housed in a pathogen-free environment under 12 h light-dark cycles with free access to water and to standard chow diet. Ten-12-week-old male and female mice were injected into the peritoneal cavity with 1x10^11^ viral genomes per 150 uL of an adeno-associated virus type 1 (AAV1) expressing mouse LDL-receptor shRNA (shLdlr) or a scramble shRNA (shSCR) (Table 1). Mice were euthanized with isoflurane (2%) and CO_2_ two weeks later and perfused with PBS. The liver, aorta and popliteal lymph nodes (LN) were harvested and processed for the assessment of LDLR protein (flow cytometry and western blot) or mRNA (qPCR) expression. All experiments were performed in accordance with the Canadian Council on Animal Care guidelines and approved by the Montreal Heart Institute Animal Care Committee (protocol #2019-14-02).

### Measurement of lymphatic function *in vivo*

Migration of dendritic cells to LN was evaluated following a contact sensitization assay 16. Briefly, the animals were sacrificed 18 h after an epicutaneous application of a solution containing fluorescein isothyiocyanate (FITC) (Sigma Aldrich cat. #F7250-250MG), dibutyl phtalate and acetone (Sigma Aldrich), and corresponding skin-draining LNs were collected and enzymatically digested in collagenase D (Cedarlane cat. #11088882001) for 25 min at 37 °C. Cells were passed through a 70 µm strainer, washed, counted and stained for analysis by flow cytometry (BD FACSCelesta^TM^). The following fluorescence-conjugated antibodies were used: anti-mouse CD11b PerCp-Cy5.5 (BioLegend cat. #101228), anti-mouse CD11c BV786 (BD Biosciences cat. #563735) and anti-mouse MHCII PE (Tonbo Biosciences cat. #35-5321-U100). The percentage of FITC-positive dendritic cells that migrated to the corresponding draining lymph nodes was analyzed with FlowJo™ software (Tree Star Inc.).

### *In vivo* measurement of lymphatic vessel contraction

Mice were anesthetized with isoflurane (2%) and oxygen (2%), placed in abdominal decubitus on a heat pad at 40 °C and the contraction capacity of the popliteal lymphatic measured as previously described 15, 28, with minor modifications. Briefly, 10 µL of Alexa-Fluor 647-conjugated ovalbumin (2 mg/mL, Thermofisher Scientific cat. #O34784) was injected in the dermis of the footpad followed by three manual contractions of the leg and a smooth massage of the injection site. After 5-10 min, the lymphatic contractions were recorded for 10 min with the Axiozoom V.16 microscope (Zeiss). A total of 326 pictures per imaging session were taken with an exposure time of 1 s. Images were stabilized using the Template Matching Imagej™ plugin and analyzed with the LymphPulse 3.0 Matlab™ based software (Figure S1). Five regions of interest (ROI) were manually sketched on each lymphatic vessel and the background intensity was subtracted. The projected curves displayed peaks and valleys with a detection threshold placed at 40 arbitrary units (AU) by the user. The contraction frequency was then computed from each curve with a Matlab algorithm following the formula (Np+Nv)/(2*dt), where Np et Nv are the number of peaks and number of valleys, respectively, and dt is the analysis time. The contraction amplitude was calculated as the mean fluorescence intensity (MFI) change within each contraction out of the maximum fluorescence intensity reached from each ROI and expressed in percentage.

### Cell culture

Primary human dermal lymphatic microvascular endothelial cells from adult donors (HMVEC-dLyAd), called human lymphatic endothelial cells (LEC) throughout the manuscript, were cultured according to the manufacturer's protocol (Lonza cat. #CC-2810) in EBM-2 medium containing the EGM-2 MV SingleQuots (PromoCell) in 5% CO_2_ atmosphere at 37 °C. Cells were seeded in 6-well cell culture plates, incubated in FBS-free media for 1 h and treated with either human recombinant PCSK9 or vehicle control in FBS-free media (6.5 ug/mL, ProSci cat. #96-577) for 16 h or transfected with human LDLR siRNA (25 nM, Qiagen Hs_LDLR_2 cat. #SI00011172 or Hs_LDLR_3 cat. #SI00011179) or non-targeting siRNA (Qiagen cat. #1027280) for 48 h in EGM-2 MV using TransIT-X2® Dynamic Delivery System (MirusBio cat. #MIR6003). Human hepatoma cell lines HepG2 and Huh7 cells and HEK 293T cells were cultured in Dulbecco's Modified Eagle's Medium (DMEM, Wisent cat. 319-005-CL) containing 10% FBS (Wisent cat. 080-350) in a 5% CO_2_ atmosphere at 37 °C. Cells were used at 70-90% confluency for all experiments. To exclude any siRNA off-target effect, two sequences were used (Table 1), herein called siLDLR2 and siLDLR3.

### Detection of lymphatic markers by flow cytometry

Human LEC (or human hepatoma Huh7) cells were harvested from the cell culture plates and centrifuged at 430 g for 5 min. All sample cells were suspended in PBS supplemented with 0.1% (vol/vol) bovine serum albumin (BSA) and 0.1% sodium azide. The following fluorescence-conjugated antibodies were used in this study: anti-human LDLR (clone ID 301, SinoBiological cat. #10231-R301-A), anti-human VEGFR-3 (clone ID 9D9F9, BioLegend cat. #356204), anti-human LYVE-1 (Invitrogen cat. #PA5-22782) and anti-human podoplanin (clone ID NC-08, BioLegend cat. #337012). Flow cytometry was performed using the BD FACSCelesta^TM^ and data were analyzed using FlowJo^TM^ software (Tree Star Inc.). Unless indicated otherwise, results are expressed as mean fluorescence intensity (MFI) and each treatment was normalized to its respective control.

### Assessment of cell cycle distribution

Human LEC were fixed in cold 70% ethanol, washed and stained with a propidium iodide (PI) solution (Biotium cat. #40017), which is fluorogenic and binds to nucleic acids in a stoichiometric manner to allow the assessment of the proportion of cells in each phase of the cell cycle (pre-replicative (G0/G1), replicative (S) and post-replicative and mitotic cells (G2/M)) 29. Flow cytometry was performed using the BD FACSCelesta^TM^ and data were analyzed using FlowJo^TM^ software (Tree Star Inc.).

### Measurement of LDL internalization

LDL internalization by human LEC was determined by flow cytometry (BD FACSCelesta^TM^ ) following a 4 h incubation with 10 μg/mL of Dil-LDL (Alfa Aesar™ cat. #J5330AMH). Unless indicated otherwise, results are expressed as mean fluorescence intensity (MFI) and each treatment was normalized to its respective control.

### Quantification of extracellular vesicle production in cell culture media

Following the 48 h treatment with siLDLR (or siControl), the cell culture supernatant was collected and the concentration of human LEC-derived extracellular vesicles (EV) determined by flow cytometry (BD FACSCelesta^TM^). For EV analysis, the 450/40 bandpass filter (BV421, violet laser) was manually swapped after cytometer setting and tracking (CS&T) calibration with a 1 mm-thick magnetron sputtered 405/10 bandpass filter (Chroma Technology, Bellows Falls, VT, USA), which is referred as V-SSC in this manuscript. Plots and histograms are showing all parameters in height (indicated as -H), as recommended for EV detection 30. Events were acquired at a flow rate of 12 µL/min, which is the lowest flow rate on the FACSCelesta. The flow rate during acquisition was kept to the minimum to avoid swarming effects and coincident detection 31. For a more precise calibration for assessment of biological vesicle size (refractive index in the range 1.36 to 1.42), the flow cytometer was calibrated for EV detection using the ApogeeMix (#1493, Apogee Flow Systems, Hemel Hempstead, UK), which consists of a mixture of non-fluorescent silica beads (180, 240, 300, 590, 880, and 1300 nm) and FITC-fluorescent latex beads (110 and 500 nm) (Figure S2). The EV gate was set to contain events ranging from ~100 to ~1000 nm using the size of the non-fluorescent silica beads in the ApogeeMix, whose refractive index is close to that of cellular membranes 32. The threshold for the forward scatter (FSC) detector was set at the lowest possible value (200 V) in FACS Diva software (BD Biosciences). EV were stained with CFSE (esterase activity, eBioscience cat. #65-0850-84) 33 and antibodies against CD31 (clone ID WM59, BD Biosciences cat. #563653) and VEGFR-3 (clone ID 9D9F9, BioLegend cat. #356204). The background in the EV gate was determined by running samples containing all reagents and antibodies except EV-containing culture media and was subtracted from the values obtained for samples with EV-containing culture media. To confirm the cellular origin of the vesicles detected, 0.1% Triton X-100 (0.05% final concentration) was added to the samples for 30 min, and the decrease in EV count denoted. The absolute concentration of EV/mL was calculated using count beads (#1426, Apogee Flow System) and the formula: (# of events in EV gate/# of events in count beads gate)*(total number of beads in sample/sample volume)*dilution factor. Data were analyzed using FlowJo^TM^ software (Tree Star Inc.).

### Immunofluorescence

LDLR expression was also determined by immunofluorescence on human LEC seeded in glassbottom culture dishes chamber (MatTek). Live cells were stained for 30 min at 37 °C with anti-human LDLR Alexa Fluor 647 Conjugate (Bioss cat. #bs-0705R), Cholera Toxin Subunit B Alexa Fluor 555 Conjugate (Invitrogen cat. #C-34776) and nuclei marker DAPI (4',6-diamidino-2-phenylindole, BioShop cat. #DAP444.5). Live images were acquired at 37 °C, 5% CO_2_ with a LSM 710 Confocal Microscope (Zeiss) equipped with a 63/1.4 oil DIC objective. Z-stacks were deconvolved with Huygens Professional (Scientific Volume Imaging, SVI, Netherlands) using a theoretical spread function (PSF). Three-dimensional rendering and quantitative colocalization (whole volume) analysis of LDLR and cholera toxin was estimated (Colocalization Analyzer, SVI) using Pearson's correlation coefficient, for which an absolute value of one indicates a perfect linear relationship, whereas a correlation close to 0 indicates no linear relationship between the variables.

### Immunoblotting

Proteins were extracted from either human LEC, human HEK 293T, human liver cell lines (HepG2 or Huh7) or mouse tissues (liver or aorta) using ice-cold radioimmunoprecipitation (RIPA) assay buffer and the protein concentration of the supernatants were established using MicroBCA™ Protein Assay Kit (ThermoFisher). Protein samples were diluted in 4X Laemmli buffer, heated at 95 °C for 5 min, separated by electrophoresis on a 10% SDS-PAGE and then transferred on a polyvinylidene fluoride (PVDF) membrane for 90 min at 4 °C. The membranes were blocked with 5% nonfat dry milk in Tris-buffered saline (TBST, 0.1% Tween 20) for 1 h at room temperature. PVDF membrane containing protein isolated from the various cell lines were then incubated overnight at 4 °C with anti-human PCSK9 (Abcam cat. #AB95478), anti-human LDLR (R&D Systems cat. #AF2148-SP), or anti-human beta-actin (Abcam, AB8227). For mouse tissues, anti-mouse LDLR (RD Systems, cat. #AF2255) and anti-mouse GAPDH (Invitrogen cat. #AM4300) were used. The membranes were washed with TBST and incubated with horseradish peroxidase (HRP)-conjugated secondary antibodies (Abcam, AB6721 or AB6741) for 90 min at room temperature. A Clarity™ Western ECL Blotting Substrates Kit (Thermofisher cat. #PI32209) was used for detection.

### ELISA

PCSK9 was measured according to the manufacturer's instructions (Human Proprotein Convertase 9/PCSK9 Quantikine ELISA Kit) (R&D Systems cat. #DPC900) in human LEC, HuH7 and HEK 293T supernatants that were concentrated using the Amicon® Ultra-2 centrifugal filter device (Milipore).

### Messenger RNA analysis by RT-qPCR

Treated cells were harvested, suspended in RiboZol™ RNA Extraction Reagent (VWR cat. #CA97064-950) and stored at -80 °C for at least 24 h. The RNA was extracted using the PureLink RNA Mini Kit extraction kit (Invitrogen cat. #12183018A) according to the manufacturer's protocol and quantified on a NanoDrop™ 1000 Spectrophotometer (ThermoFisher). Reverse transcription of RNA was performed using the iScript kit cDNA synthesis kit (Thermofisher cat. #4368814). Quantitative PCR was performed on the QuantStudio™ 3 (ThermoFisher Scientific) using 10 ng/well of complementary DNA (cDNA) mixed with iTaq™ Universal SYBR Green Supermix (Biorad cat. #1725121). The primers used are displayed in Table 1. The amplification cycles were performed at 94 °C for 10 s and at 60 °C for 45 s. The relative expression was evaluated using the comparative method (2-ΔΔCT) and normalized to the control gene.

### Transcriptomic analysis

Samples were submitted for Illumina next-generation sequencing to the IRIC Genomics Platform. Adaptor sequences and low-quality bases in the resulting FASTQ files were trimmed using Trimmomatic version 0.35 34 and genome alignments were conducted using STAR version 2.7.1a 35. The sequences were aligned to the human genome version GRCh38, with gene annotations from Gencode version 37 based on Ensembl 103. As part of quality control, the sequences were aligned to several different genomes to verify that there was no sample contamination. Gene expressions were obtained both as raw readcount directly from STAR as well as computed using RSEM 36 in order to obtain gene and transcript level expression in reads per transcripts per million (TPM) for these stranded RNA libraries. DESeq2 version 1.22.2 37 was then used to normalize gene readcounts and compute differential expression between the various experimental conditions. Sample clustering based on normalized log read counts produces a hierarchy of samples.

### Non-targeted lipidomic by liquid chromatography quadrupole time-of-flight mass spectrometry (LC-QTOF-MS)

The LC-QTOF-MS was performed by the Montreal Heart Institute Metabolomics core facility as previously described 38. Cells were seeded in 60 mm glass petri dishes (Pyrex™ cat. #C316060) to avoid polymer (polyethylene glycol, PEG) contamination, and treated as described above. Cells were then harvested, washed, and suspended in 1 mL of serum-free medium. Lipids were extracted following the addition of internal standards (monoacylglycerosphosphocholine (LPC) 13:0, diacylglycerophosphocholine (PC) 14:0/14:0 and 19:0/19:0, phosphatidylserine (PS) 12:0/12:0, diacylglycerophosphoethanolamine (PE) 17:0/17:0, and diacylglycerophosphoglycerol (PG) 15:0/15:0)) and injected into a high performance liquid chromatograph (1290 Infinity HPLC) coupled to quadrupole time-of-flight mass spectrometry (Agilent Technologies Inc.) equipped with a dual electrospray ionization source and analyzed in positive and negative mode. The lipid elution was carried out on a Zorbax Eclipse plus column (C18, 2.1 mm × 100 mm, 1.8 μm, Agilent Technologies Inc.) for 83 min at a constant temperature of 40 °C in a gradient of solvent A (0.2% Methanoic acid and 10 mM ammonium formate in water) and B (0.2% methanoic acid and 5 mM ammonium formate in methanol/acetonitrile/methyl tert-butyl ether [MTBE], 55:35:10 [v/v/v]). Mass spectrometry data analysis was performed with the Mass Hunter Qualitative Analysis software (B.07 version) and discriminant lipids were identified by Tandem Mass Spectrometry (MS/MS). Statistical analyzes were carried out by unpaired Student's t-test followed by Benjamini-Hochberg correction with the program Mass Professional Pro version 12.6.1 (Agilent Technologies Inc.).

### Cholesterol measurement

Blood was collected on 100 mM ethylenediamine tetraacetic acid (EDTA) by cardiac puncture and plasma was obtained from wt and *Pcsk9^-/-^* mice following centrifugation at 2400 g for 10 min and were stored at -80 °C. Mouse lipoproteins were separated from plasma by size exclusion chromatography [fast protein LC (FPLC)] using a Superose 6 column on a FPLC system with a Model 500 pump from Waters (Milford, MA). In short, a 100 μL aliquot of mouse plasma pooled equally from four (4) different mice was injected into a 1.0-mL sample loop and separated with PBS at a flow rate of 0.5 mL/min. Sixty-five fractions of 0.3 mL each were collected with the lipoproteins being contained within. Batch analysis was performed to measure circulating total cholesterol (Fujifilm Medical Systems U.S.A., Inc. cat. #999-02601) in plasma and plasma lipoprotein fractions according to the manufacturer's protocol. For the *in vitro* experiment, total cholesterol measurement was performed according to the manufacturer's protocol (Total Cholesterol E, Fujifilm Medical Systems U.S.A., Inc. cat. #999-02601) in cell culture media as well as in human LEC-derived EV (elicited with Triton X-100) following the 48 h treatment with LDLR siRNA or non-targeting siRNA.

### Statistics

Data are expressed as mean ± standard error of the mean (SEM). Statistical significance was evaluated by unpaired t test or, for multiple comparisons, one-way ANOVA using appropriate corrections when data was not normally distributed, or for unequal variances. Each *in vitro* experiment was performed in triplicate. All calculations were done with GraphPad Prism v8 software (GraphPad Software, La Jolla, CA, USA), and p-values <0.05 were considered statistically significant.

## Results & Discussion

Acute exposure of collecting lymphatic vessels to LDL in mice increases contraction frequency and lymph flow 39. Therefore, LDL binding to LDLR could appear as an attractive target in preserving lymphatic function. However, lymph is rich in high-density lipoprotein (HDL) and chylomicron, but rather comparatively poor in LDL 40, which rises the concern on the physiological relevance of this previous finding. Furthermore, LDLR deficiency has been associated to an early lymphatic dysfunction in pre-atherosclerotic mice before major changes in circulating lipid levels 16. The injection of VEGF-C 152S before the administration of a pro-atherogenic regimen improves lymphatic contraction capacity in *Ldlr^-/-^* mice and protects them from excessive plaque formation 15. Therefore, we hypothesize that LDL binding to LDLR on lymphatic endothelial cells levels would only have a minor role, if any, in maintaining lymphatic contraction capacity in the initial steps of atherosclerosis. We thus sought to determine whether and how lymphatic endothelial-LDLR might modulate lymphatic function.

### Decreasing *Ldlr* expression in lymphatic endothelial cells does not alter plasma lipoprotein distribution nor dendritic cell transport through lymphatics in 3-month-old mice

**Figure 1:**
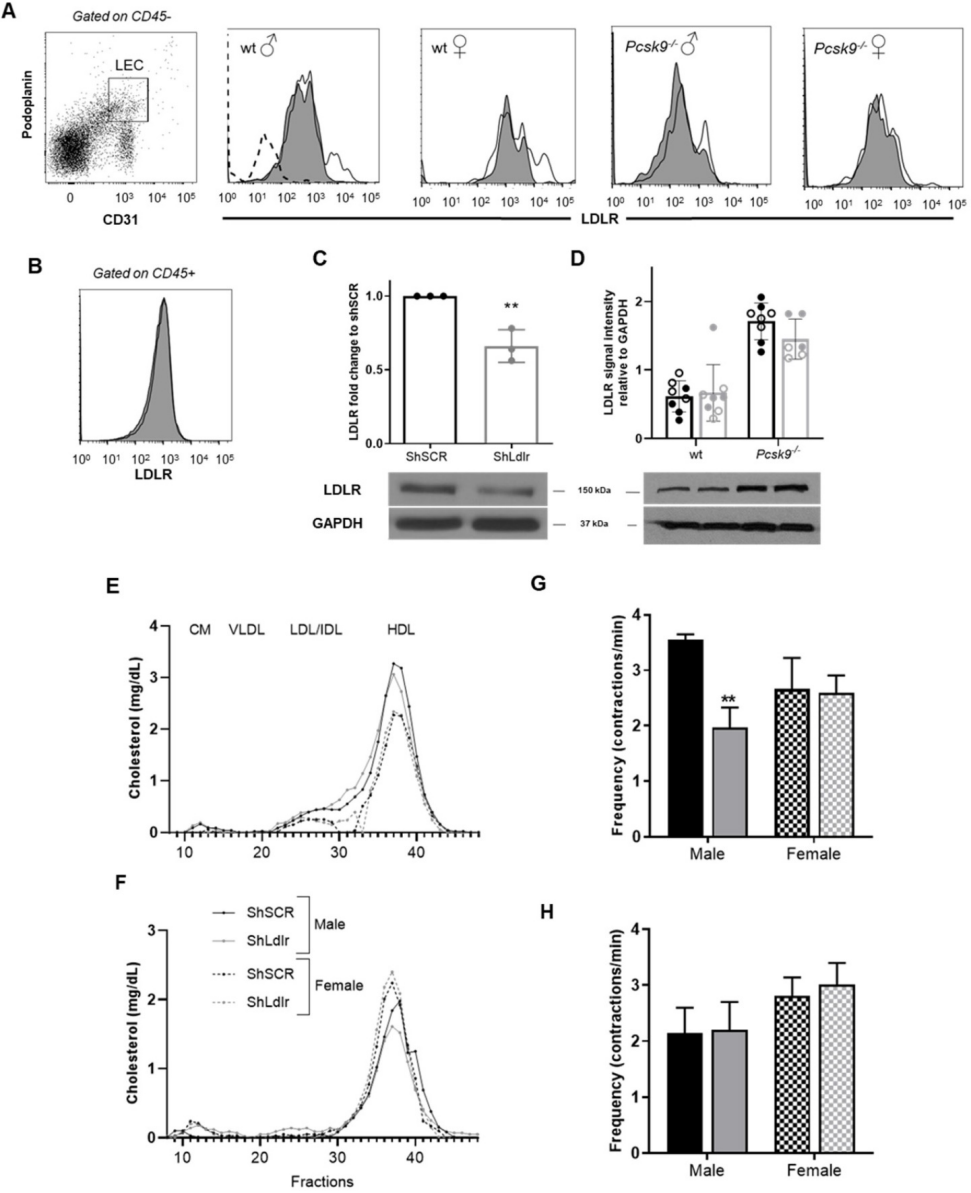
** Lymphatic function assessment in mice following a specific knockdown of LDLR in murine endothelial cells.** Two weeks after the intraperitoneal injection of an adeno-associated virus type 1 (AAV1) containing a shRNA targeting LDLR, skin draining lymph nodes were harvested, digested and analyzed by flow cytometry to assess lymphatic endothelial cell (LEC)-specific knockdown of LDLR expression in mice. Membrane-bound LDLR expression was measured on **(A)** CD45^-^CD31^+^Podoplanin^+^ and **(B)** CD45^+^ cells. Fluorescence minus one (FMO) control was used for LDLR expression, as depicted by the dotted line in the first histogram. White histogram, shSCR and dark histogram, shLldlr. LDLR expression was also determine by immunoblotting in **(C)** aorta isolated from female mice and **(D)** in liver from wild-type and *Pcsk9^-/-^* male (full dot) and female (empty dot) mice. ShSCR in black and ShLdlr in grey. Total plasmatic cholesterol was measured following FPLC in each liproprotein subfraction in **(E)** wild-type and **(F)** *Pcsk9^-/-^* female (dotted lines) and male (solid lines) mice treated with shSCR (black lines) and shLdlr (grey lines). Lymphatic contraction capacity was assessed by fluorescent *in vivo* imaging in **(G)** wild-type and **(H)** *Pcsk9^-/-^* mice. Black histogram, ShSCR and grey histogram, ShLdlr. n=3-9. Statistics **p < 0,01.

*Pcsk9*^-/-^ mice display improved dendritic cell migration through the lymphatics in 6-month-old mice, but not in 3-month-old mice, compared to age-matched wild-type animals 16. As lymph and plasma PCSK9 levels are increased in LDLR-deficient mice compared to control mice 16, we have first tested whether PCSK9 is essential in disrupting lymphatic transport in the absence of LDLR *in vivo*. We have injected 3-month-old *Pcsk9^-/-^* or wild-type mice with an adeno-associated virus type 1 (AAV1) expressing mouse LDL-receptor shRNA (shLdlr) or a scramble shRNA (shSCR) sequence (Table 1). The AAV1 containing the shLdlr efficiently decreased LDLR expression in endothelial cells as revealed by flow cytometry (Figure 1A, see Figure S3 for gating strategies) and immunoblotting (Figure 1C) whereas it had no effect in the liver (Figure 1D) or lymph-node CD45^+^ cells (Figure 1B). We have already published that *Pcsk9^-/-^* mice have more membrane LDLR-LEC than their wild-type controls, as represented by immunofluorescence 16. Immunoblotting show that total LDLR protein levels in the liver were higher in *Pcsk9^-/-^* mice compared to wild-type animals 41 (Figure 1D). Decreasing LDLR levels on endothelial cells had no effect on the plasma lipoprotein distribution in wild-type or *Pcsk9^-/-^* mice (Figures 1E and 1F, respectively). This excludes that circulating cholesterol might act as a major contributor of the modulation of lymphatic function when LDLR is downregulated. Furthermore, neither *Pcsk9*^-/-^ nor wild-type mice had significant changes in dendritic cell transport within lymphatic vessels (Figures S4A-B, see Figure S4C for gating strategies) 16 following shLdlr or shSCR injection. This implies that there is no apparent impairment in lymphatic transport yet in these 12-week-old mice.

### PCSK9 is needed to reduce lymphatic contraction capacity in endothelial-specific LDLR knockdown mice

Mice deficient in *Ldlr* display an impaired lymphatic contractility at 12-week-old, prior to a disrupted lymphatic transport and atherosclerotic plaque formation 15, 16, 42. Consequently, we thus next investigated whether a failure in the contraction capacity could also be observed in mice treated with shLdlr. Using intravital imaging, the capacity of the popliteal collecting lymphatic vessel to contract was evaluated following the injection of fluorescent ovalbumin (Figure S1). Knocking-down LDLR expression in lymphatic endothelial cells significantly decreased the number of contractions per minute observed in wild-type male mice (Figure 1G). It had no effect in wild-type females (Figure 1G), despite its efficiency to decrease total endothelial- LDLR expression (Figures 1A and C). Contractions also remained unchanged in *Pcsk9*^-/-^ mice, of both sexes (Figure 1H). Thus, PCSK9 seems to be needed to reduce lymphatic contraction capacity in endothelial-specific LDLR knockdown mice, nonetheless in a sex-specific manner. It could partially be explained by the more modest decrease in membrane-LDLR observed following shLldlr injections in these mice (Figure 1A, shift of the histogram toward the left), concurring with studies affirming that the absence of PCSK9 results in a sex- and tissue-specific subcellular distribution of the LDLR 41. *Ldlr* mRNA levels have also been described as less sensitive to estrogen hormone variations in mice lacking PCSK9 41, which might be a cause of the differences we herein observe between wild-type and *Pcsk9*^-/-^ male and female mice. Circulating PCSK9 interacts with reactive oxygen species (ROS) and promotes vascular aging and atherosclerosis progression 43. ROS production is closely linked to nitric oxide (NO) generation and elevated levels of ROS lead to low NO bioavailability, as observed in mice that are systemically lacking LDLR 44. Like in blood vessels, NO is a fine regulator of lymphatic function, mainly through the regulation of collecting lymphatic vessel contractions 45. ROS negatively influences lymphatic contractile function 46, and under inflammatory conditions, lymphatic contraction frequency is reduced due to the increase secretion of NO from the inducible nitric oxide synthase (iNOS) 47. One of the major driving forces of lymphatic pumping is the surrounding layer of muscle cells whose activity largely depends on intracellular Ca^2+^ levels 48. Ca^2+^ levels are modulated by NO levels produced by the endothelial NOS (eNOS) under physiological conditions. Estrogen receptor alpha (ERα) signaling has recently been suggested to protect from lymphatic dysfunction 49. Besides, the estradiol (E2)/ERα specific binding regulates the expression of several genes, including *Ldlr* 50 and endothelial NOS (*enos*) 51. The reduced estrogen production in the female mice used in our study compared to age-matched male mice 52 might limit nitric oxide (NO)-dependent contractile forces in the lymphatic endothelium.

### Human lymphatic endothelial cells do not express nor secrete PCSK9

**Figure 2:**
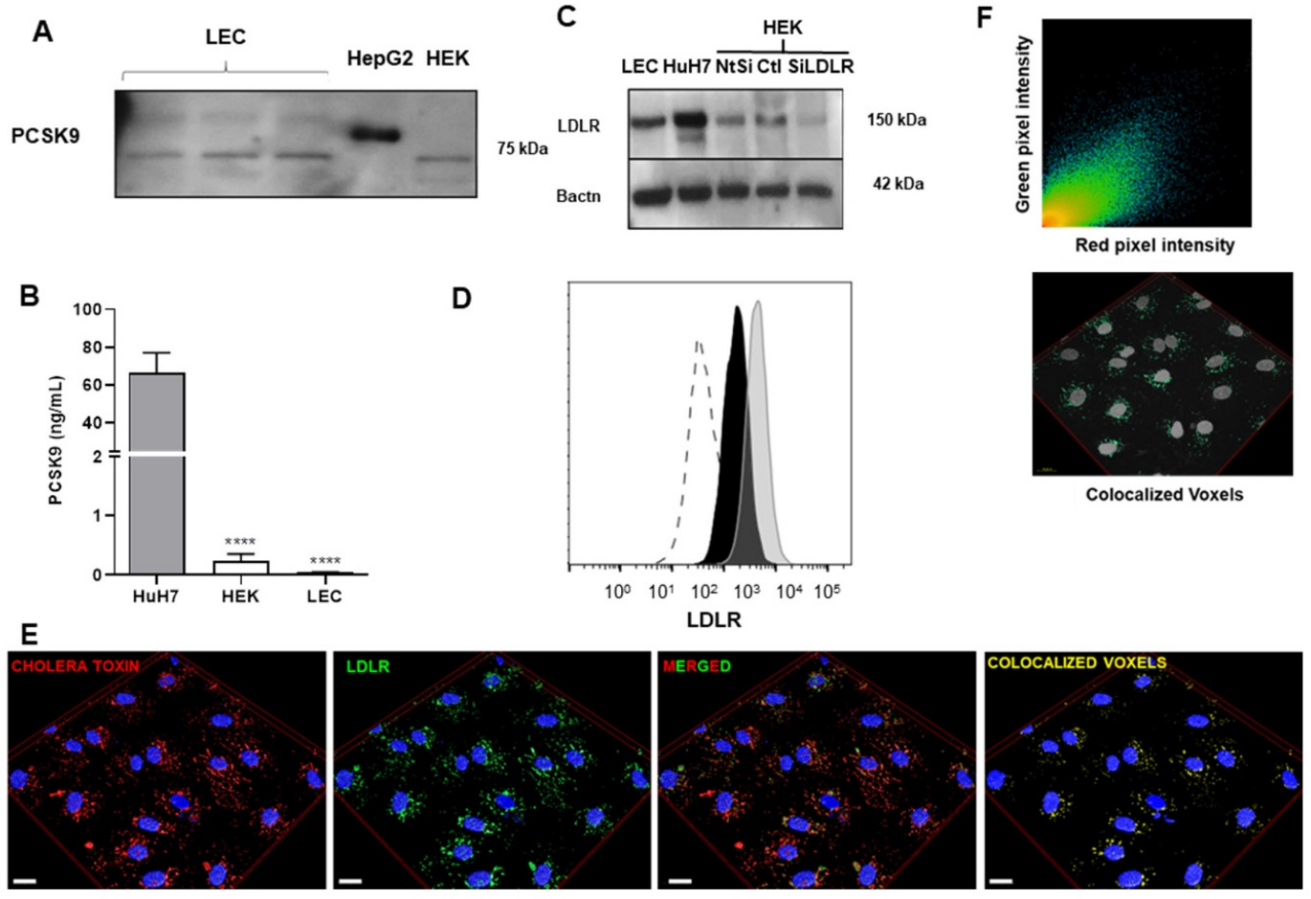
** Assessment of PCSK9 and LDLR expression in human LEC. (A)** PCSK9 expression was measured by immunoblotting in either human LEC, HepG2 or HEK 293T cell lysates. **(B)** ELISA was used to measure PCSK9 levels in the cell culture supernatant of Huh7 (grey), HEK 293T (white) and human LEC (black). **(C)** Expression of LDLR was detected in protein lysates by immunoblotting of human LEC. Huh7 cells were used as a positive control and HEK 293T cells treated with siLDLR were used as a negative control. LDLR protein expression on human LEC was measured by **(D)** flow cytometry after extracellular staining of LEC (black line) and Huh7 (grey line) and by **(E)** immunofluorescence (Blue, DAPI; red, cholera toxin; green, anti-LDLR; yellow, colocalized voxels; scale bar, 20 µM). **(F)** Scatterplot of red and green pixel intensities of cholera toxin (red) and anti-LDLR (green) in human LEC. n = 4-9. Statistics: ****p < 0.0001.

We then sought to investigate in further details how PCSK9 and LDLR can affect lymphatic function at the cellular level. As PCSK9 enhances pro-inflammatory reactions in the vascular wall 53-56, we hypothesized that PCSK9 might trigger similar effects on lymphatic endothelial cells. Our laboratory has demonstrated that PCSK9 circulates in mouse lymph and that lymphatic function is improved in 6-month-old *Pcsk9*^-/-^ mice 16. However, the source of PCSK9 in lymph remains unknown. We herein show that lymphatic endothelial cells do not express (Figure 2A) nor secrete (Figure 2B) PCSK9. Therefore, LEC, like other cell types that do not produce PCSK9 22, are probably elicited by circulating PCSK9 produced mainly by the liver, and retrieved in lymph 16.

### LDLR is localized in lipid rafts in human lymphatic endothelial cells

PCSK9 is typically recognized for its role in the regulation of cholesterol metabolism; it binds hepatic LDLR and induces its degradation in lysosomes leading lower cell surface LDLR and to an increase in plasma LDL-cholesterol 57. In the past decade, PCSK9 has been studied for its role in atherosclerosis through pleiotropic effects on diverse cell types 58-60. It has been reported that circulating PCSK9 contributes directly to the progression of atherosclerosis by enhancing blood endothelial cells dysfunction independent of its effect on the LDLR 61. We herein sought to define whether PCSK9 can control LDLR expression or if it rather acts through an LDLR-independent mechanism. We have previously demonstrated that LDLR is expressed on mice LEC, but not on lymphatic muscle cells 16. Our second step *in vitro* was consequently to highlight whether and how LDLR is expressed on human LEC. The expression of LDLR was first confirmed by immunoblotting in whole cell lysates of human LEC, HepG2 and HEK 293T (Figure 2C). Flow cytometry allowed for the detection of membrane LDLR on human LEC (Figure 2D). Immunofluorescence images revealed that LDLR is mostly likely located in the lipid rafts (Figure 2E), as it is positively correlating with the presence of GM1 gangliosides labeled by cholera toxin subunit B (Pearson coefficient, 0.804; Figure 2F) within the cells. Interestingly, on other cell types, LDL binds to the LDLR in clathrin-coated pits, rather than LDLR-associated with lipid rafts 62. Our finding highlights the unique regulation of LDLR in LEC and suggests that the binding of ligands other than LDL modulates its expression.

### LDLR expression remains unchanged upon LDL internalization by human lymphatic endothelial cells

**Figure 3:**
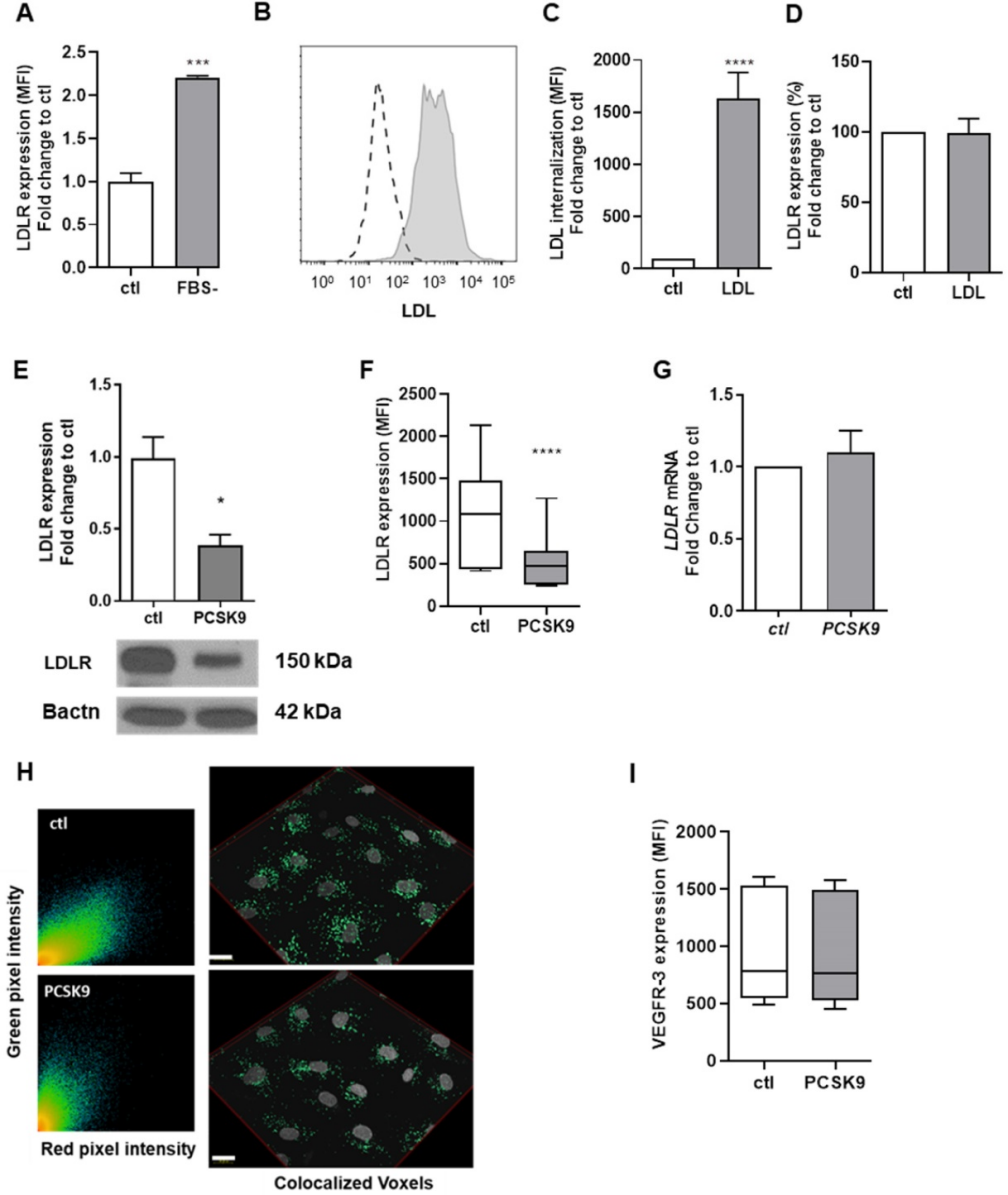
** LDLR regulation in human LEC. (A)** Cell surface expression of LDLR was measured by flow cytometry after incubating human LEC with (ctl) or without FBS-containing media for 24 h. **(B)** Human LEC were incubated with Dil-LDL for 4 h and LDL uptake was measured by flow cytometry (dotted line, no LDL; grey line, Dil-LDL). **(C)** The percentage of LDL internalization compared to control as well as **(D)** cell surface expression of LDLR was then quantified by flow cytometry. Human LEC were incubated with exogenous human recombinant PCSK9 (6.5 µg/mL) for 16 h and LDLR protein expression was detected and quantified by **(E)** immunoblotting,**(F)** flow cytometry and **(G)** *LDLR* messenger RNA by qPCR **(H)** Immunofluorescence confirmed LDLR expression in human LEC. Representative scatterplot of red and green pixel intensities of cholera toxin subunit B (red) and anti-LDLR (green) in lymphatic endothelial cells and a reconstruction of colocalized voxels (scale bar, 20 µM). **(I)** Protein expression of VEGFR-3 was measured in human LEC by flow cytometry following a 16 h incubation with 6.5 µg/mL human recombinant PCSK9. n=3-9. Statistics: *p < 0.05, ***p < 0.001, ****p < 0.0001.

To further study the physiological role of LDLR-LEC, we incubated human LEC with fetal bovine serum (FBS)-free media for 24 h and cell surface expression of LDLR was measured by flow cytometry. Decreasing surrounding lipid content increased LDLR levels on the cell membrane (Figure 3A). However, when incubating human LEC with dil-LDL, LEC could efficiently internalize LDL (Figures 3B-C) despite no changes in membrane LDLR, as determined by flow cytometry (Figure 3D). Therefore, LDLR modulation on LEC could have completely different physiological functions than on any other cell type and affect lymphatic function in a mechanism that is independent of its LDL binding properties. Apolipoprotein (apo) B and apoE-containing particles other than LDL might bind to LDLR, which could mediate their internalization in LEC. This mechanism remains to be tested.

### Exogenous PCSK9 decreases LDLR protein but not *Ldlr* mRNA expression in human lymphatic endothelial cells

As hepatic and extra-hepatic cells such as fibroblasts are also known to overexpress LDLR when incubated with serum-depleted medium 63, 64, we then investigated whether LDLR levels on LEC would also be modifiable through the action of PCSK9. We incubated human LEC for 16 h with recombinant PCSK9 (6.5 μg/mL) and observed a decrease in total LDLR protein by immunoblotting (Figure 3E) and a 90% decrease of the membrane-bound LDLR protein by flow cytometry (Figure 3F). Yet, no reduction in *LDLR* mRNA was denoted (Figure 3G). Further, exogenous PCSK9 did not affect the Pearson coefficient assessing the colocalization between LDLR and cholera toxin subunit B (average of 0.694 with exogenous PCSK9 vs. 0.804 for control; Figure 3H). Our findings report that PCSK9 treatment modulates LDLR expression at the protein level but not at the mRNA level.

### Exogenous PCSK9 does not modulate the expression of lymphatic markers nor the endothelial cell cycle

Signaling by vascular endothelial growth factors (VEGFs) through VEGF receptors (VEGFRs) plays important roles in vascular development 65. VEGFs bind to the three known VEGFRs, VEGFR-1, VEGFR-2 or VEGFR-3, which are expressed by blood vessel endothelial cells in case of VEGFR-1 and -2, and LEC in case of VEGFR-2 and -3. Specific activation of VEGFR-3 by VEGF-C 156S, a selective VEGFR-3 agonist, stimulates lymphatic pumping by a VEGFR-3-dependent mechanism 66 and downregulates many genes involved in immune regulation and inflammation 67, suggesting that VEGFR-3 stimulation has direct anti-inflammatory effects. Furthermore, lymphatic transport can be restored in *Ldlr^-/-^* mice by systemic injections of an analogue of VEGF-C 156S, namely VEGF-C 152S. We have herein demonstrated *in vivo* that PCSK9 might be a key contributor of lymphatic contraction and function impairment, but the underlying mechanisms remain to be investigated. PCSK9 inhibitors have been shown to increase VEGFR-2^+^ endothelial progenitor cells 68 and increase VEGF release into supernatants of blood endothelial cells *in vitro* 69. We herein wanted to assess whether PCSK9 treatment would decrease VEGFR-3 expression on human LEC. Albeit it decreased LDLR protein expression (Figures 3E-F) without however altering the transcription of the protein (Figure 3G) 70, exogenous PCSK9 had no effect on VEGFR-3 protein expression (Figure 3I) or other lymphatic markers such as LYVE-1 (Figure S5A) or podoplanin (Figure S5B). RNA sequencing first confirmed that exogenous PCSK9 had little to no effect on gene expression (data not shown). The only gene that appeared upregulated upon PCSK9 treatment is *ATAD3C* (data not shown). ATPase family AAA domain-containing protein 3 (ATAD3C) is a mitochondrial membrane-bound ATPase involved in replication and transcription and is notably decreased in patients with hepatocarcinoma 71. Its deficiency is associated with abnormal cholesterol metabolism and mitochondrial DNA aggregation in central nervous system 72. Knowing that mitochondrial ATAD3C is involved in cellular cholesterol homeostasis and that PCSK9 downregulates LDLR expression and exogenous cholesterol uptake by LECs, its upregulation is expected.

Cholesterol synthesis is tightly related to cell proliferation 73, 74. In general, proliferating cells show increased cholesterol synthesis and LDL receptor activity, which reflects the cellular lipid storage 75. Contrariwise, cholesterol synthesis inhibitors can block cell proliferation 76-79. Suppressing PCSK9 induces apoptosis and significantly alters the cell cycle of human keratinocytes, increasing the percentage of cells in S phase compared to control treatment 80. To explore whether PCSK9 could exert its effect on lymphatic function by altering lymphatic endothelial cell cycle, human LEC were labeled with propidium iodide and the DNA content was measured to determine the proportion of cells in each phase of the cell cycle (Figure S5C) 29. Our data revealed that exogenous PCSK9 did not modify the lymphatic endothelial cell cycle *in vitro* (Figure S5D). Our results thus suggest that decreasing LDLR expression at the protein level by itself might not be sufficient to alter LEC replication and integrity. Furthermore, it has been recently shown that PCSK9 is expressed in vascular smooth muscle cells and inhibits cell proliferation, thus affecting their function 81. This data could explain the discrepancies observed our *in vivo* and *in vitro* model. Indeed, *in vivo*, PCSK9 might mediate its effect on lymphatic function by directly targeting lymphatic muscle cells in a LDLR-independent manner, rather than via lymphatic endothelial cells.

### Silencing LEC-LDLR decreases VEGFR-3 expression at the protein but not at the mRNA level

**Figure 4:**
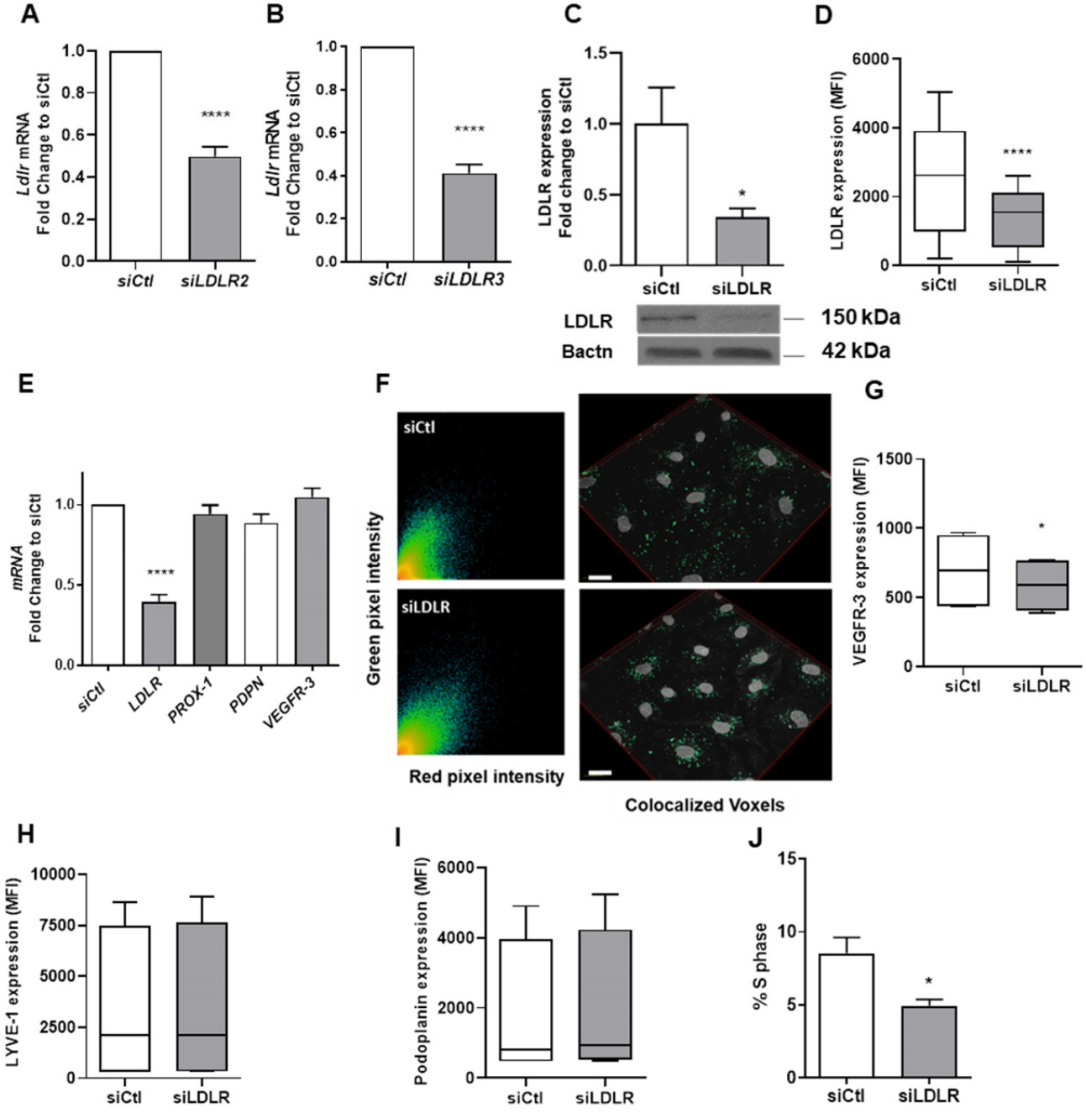
** Human LEC-*LDLR* modulation at the mRNA level.** Human LEC were transfected for 48 h with 25 nM of non-targeting siRNA (called siCtl) or siLDLR. The siRNA off-target effect of was verified by two siRNA sequences, herein called siLDLR2 and siLDLR3. Messenger RNA expression was assessed by qPCR in human LEC treated with either **(A)** siLDLR2 or **(B)** siLDLR3 or their respective control (siCtl). LDLR protein expression was measured by **(C)** immunoblotting and **(D)** flow cytometry. **(E)** *LDLR*, *PROX-1*, *PDPN (podoplanin)* and *VEGFR-3* mRNA expression in human LEC was assessed by qPCR. **(F)** Representative scatterplot of red and green pixel intensities of cholera toxin (red) and anti-LDLR (green) in lymphatic endothelial cells and a reconstruction of colocalized voxels was performed for immunofluorescence experiments (scale bar, 20 µM). Protein expression of **(G)** VEGFR-3, **(H)** LYVE-1 and **(I)** podoplanin were measured by flow cytometry. **(J)** Percentage of cells in the S phase of the cell cycle. n = 3-10. Statistics: *p < 0.05, ****p < 0.0001.

In addition to investigating the effect of a total deletion in PCSK9 on lymphatic function, our *in vivo* model also pertained to downregulation of LDLR at the mRNA level. To explore the potential cellular mechanisms that could explain the differences we observed in the contraction capacity, we have treated human LEC with a siRNA targeting LDLR (siLDLR) or non-targeting siRNA (siCtl) in complete medium. *LDLR* mRNA levels were blunted following treatment with both sequences (Figures 4A-B). The expression of LDLR protein in whole cell lysate (Figure 4C) and in the cell membrane (Figure 4D) was also efficiently lessened. Knocking-down LDLR, however, did not affect the Pearson coefficient assessing the colocalization between LDLR and cholera toxin subunit B (average of 0.688 with siLDLR vs. 0.766 for control; Figure 4F). We observed a significant decrease in the proportion of LEC in the S phase when transfected with LDLR siRNA compared to the non-targeting siRNA treatment (Figure 4J). This observation provides a strong foundation for a novel role for LDLR in mediating the lymphatic endothelial cell cycle. Unlike other lymphatic markers involved in LEC proliferation and function such as LYVE-1 (Figure 4H) and PDPN (Figure 4I), membrane-bound VEGFR-3 was reduced upon LDLR siRNA treatment (Figure 4G). Yet, VEGFR-3 mRNA levels were unchanged (Figure 4E) compared to control siRNA. The fact that VEGFR-3 is modulated by the downregulation of *LDLR* mRNA could be a key element in our comprehension of the mechanism underlying the premature defect in lymphatic transport early in the atherosclerosis process. As VEGFR-3 diminished at the protein but not mRNA level, we suggest that a reduction in membrane cholesterol, a consequence of a reduction in LDLR, could potentially increase the turnover of membrane VEGFR-3. VEGFRs are promptly internalized into endocytotic vesicles to control their activity. VEGFR-2 has been shown to colocalize with lipid rafts to regulate its activation 82. Furthermore, lipid rafts stabilize VEGFR-2 and its associated signal transduction activities required for angiogenesis 83. If VEGFR-3 makes no exception, it would mean that changes in membrane lipids could also regulate the sensitivity of lymphatic endothelial cells to VEGFR-3 stimulation.

### Silencing LEC-LDLR decreases total cellular cholesterol, cholesterol esters and key lipids involved in membrane integrity

**Figure 5:**
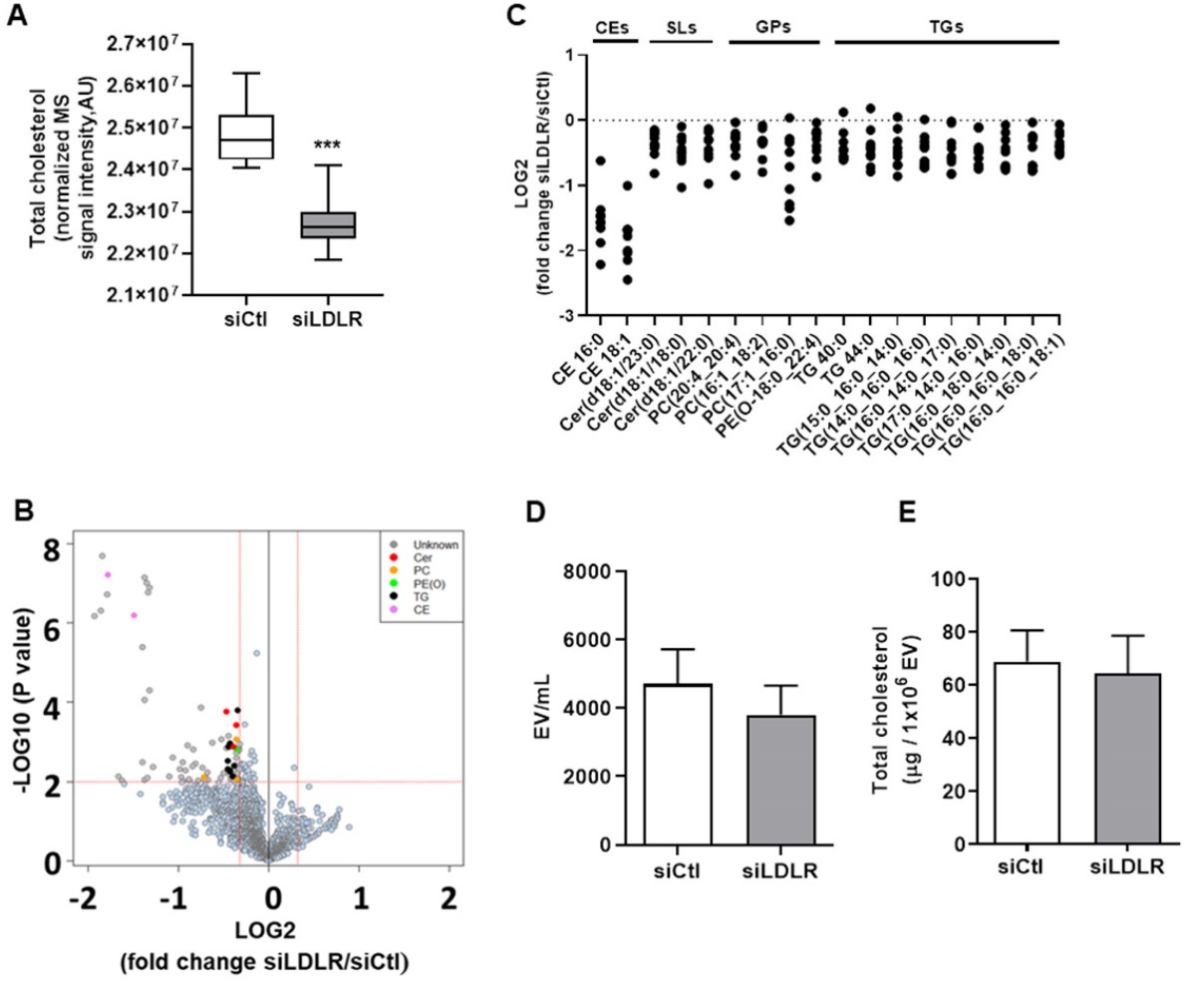
** Untargeted lipidomics in *LDLR* siRNA-treated human LEC.** Human LEC were treated *in vitro* with 25 nM of siLDLR or siCtl for 48 h and MS-based lipidomics analysis of cellular extracts from siLDLR and control-treated cells was performed. **(A)** Total cholesterol was quantified and **(B)** 2139 MS features were obtained using as criteria of selection a p-value of 0.05 (y-axis where p-values are expressed as -LOG10) and a FC of 0.8 and 1.25 (x-axis where FC are expressed as LOG2), and **(C)** 18 unique lipid (sub)classes discriminating siLDLR- and siCtl- treated cells were further identified using MSMS analysis. Each dot represents a log2-tranformed siLDLR/siCtl signal intensity ratio. **(D)** Extracellular vesicles produced by human LEC *in vitro* were quantified in supernatant by flow cytometry and **(E)** their total cholesterol content was measured following a 30 min incubation with a detergent (Triton X-100) by spectrophotometry. EV, extracellular vesicles; CE, cholesteryl esters; Cer, ceramides; GPs, glycerophospholipids; PC, phosphatidylcholine; PE(O-), phosphatidylethanolamine (plasmanylethanolamine); SLs, sphingolipids; TGs, triglycerides. “_” in TGs refers to acyl chains for which the position remains to be ascertained. n = 3-9. Statistics: ***p < 0.001, threshold P-value < 0.01.

Lipid rafts have a lifetime on the plasma membrane that ranges from seconds to minutes 84. Albeit very labile, these segregations of lipids are typically enriched in free cholesterol and glycosphingolipids and regulate important cellular functions such as cellular polarity, vesicular traffic and signaling pathways 85. Hence, we performed non-targeted lipidomic in siLDLR-treated human LEC to determine whether and how a downregulation in LDLR on LEC might change the cellular lipid composition and thus VEGFR-3 expression. First, we report that total cholesterol is decreased in cells treated with siLDLR compared to non-targeting siRNA (Figure 5A). In total, among the 2139 MS signals (or features) obtained following data processing, 80 features significantly discriminated siLDLR-treated cells from control cells (Figure 5B), among which we identified 18 unique lipids belonging to four main (sub) classes: cholesteryl esters, sphingolipids, glycerophospholipids and triglycerides (Figure 5C). Of all the lipids detected that have decreased following siLDLR treatment, phosphatidylcholines and ceramides were identified. Phosphatidylcholines are lipids which account for about 40% of phospholipids in cells 86. Their suppression in blood endothelial cells prevents normal functioning of apoptosis signaling pathways 87. Disturbance in the ratios of membrane lipids (phosphatidylcholines to phosphatidylethanolamines) leads to the development of organ failure in mice caused by loss of membrane integrity of hepatocytes 88 and affects the normal processes of cell proliferation and viability 89. Ceramides are important for the stability of the lipid bilayer 90 and they participate in the regulation of cell proliferation by inducing apoptosis under normal conditions 91, 92. In addition, we observed lower levels of CE, a decrease concurring with total cholesterol decrease. Altogether, these published data suggest that lipid changes mediated upon silencing LDLR might affect LEC integrity and function in a similar way.

### Silencing LEC-LDLR alters VEGF-C mRNA, cholesterol biosynthesis, cell cycle processes and vesicle-mediated transport

**Figure 6:**
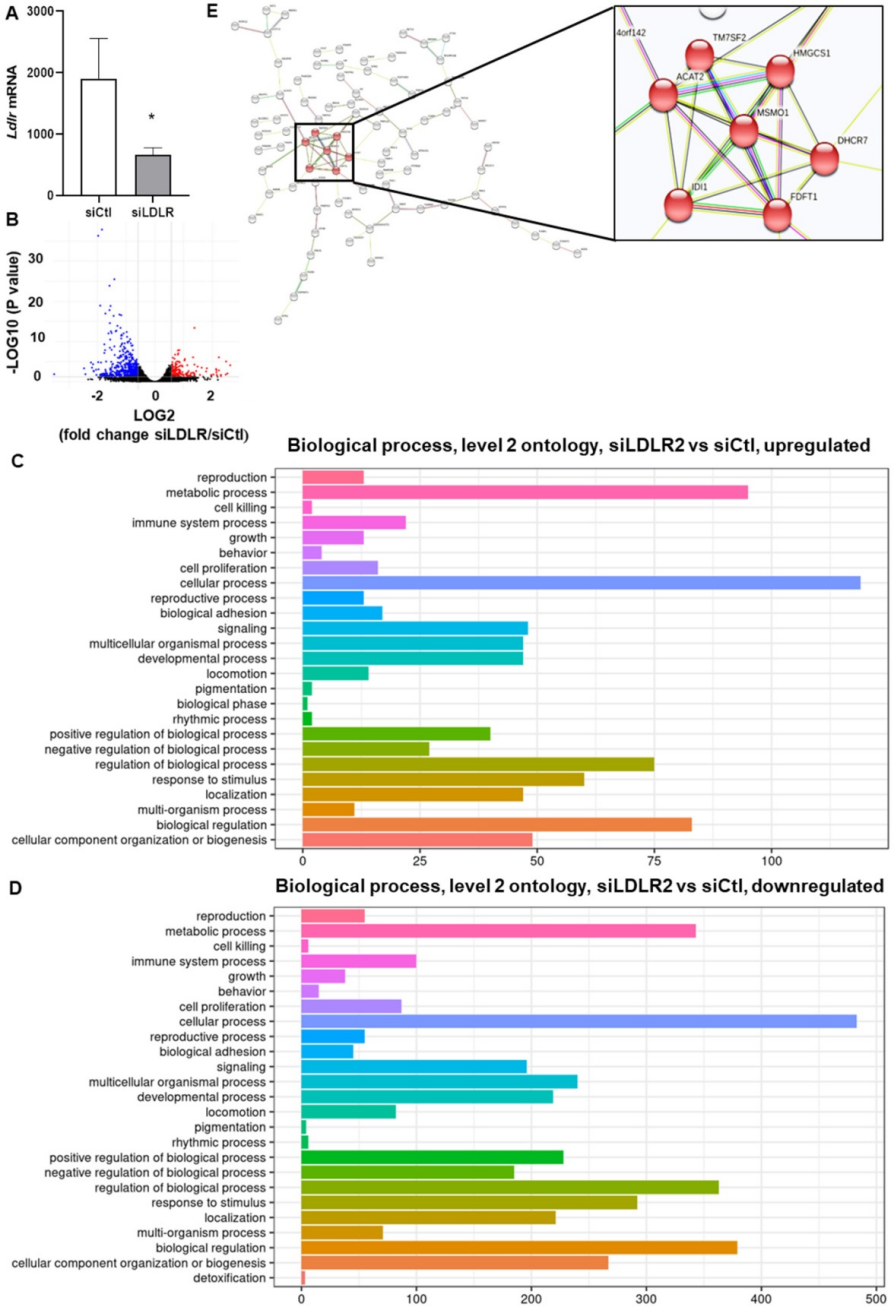
** siRNA targeting *LDLR* expression modulates genes related to cholesterol biosynthesis and cellular and metabolic processes.** Human LEC were transfected for 48 h with 25 nM of non-targeting siRNA (siCtl) or siLDLR and transcriptomic analysis was performed. **(A)** The efficiency of the treatment to decrease *LDLR* is represented. **(B)** Volcano plot of transcriptomic analysis performed in human LEC. Y axis = p value (-LOG10) of the gene expression, X axis = fold change siLDLR/siCtl (LOG2) of gene expression. Each point represents an individual gene. Significant upregulated and downregulated genes in siLDLR versus siCtl are depicted in red and blue, respectively. Using the R cluster Profiler package, function enrichment within significant genes (padj lower than 0.05 and fold change higher than 1.5), the following observed biological processes and enriched categories (using FDR adjustement to filter for 0.05 qValue cutoff) between siLDLR and siCtl was obtained. Biological processes implicated for **(C)** all the upregulated genes and **(D)** downregulated genes. **(E)** Protein-protein interaction network performed with the Search Tool for the Retrieval of Interacting Genes (STRING) database for all upregulated genes showed that cholesterol biosynthesis is the main biological process that is upregulated. n = 3. Statistics: *p < 0.05.

To explain these changes in membrane VEGFR-3 and lipids, we then sought to investigate whether siLDLR could increase the shedding of extracellular vesicles (EV) derived from the plasma membrane. Downregulating LDLR on LEC had no effect on the production of LEC-EV (Figure 5D). As we could not exclude that the EV production rate might remain unchanged but its composition different, we next measured the total cholesterol content of these EV. We show that cholesterol-EV is also unchanged compared to the control-treated group (Figure 5E), suggesting that plasma membrane cholesterol nor VEGFR-3 are not mobilized out of the cell through the production of extracellular vesicles. Transcriptomic analysis thus became insightful to better understand the potential signaling pathways involved in the alteration of lymphatic integrity following LDLR siRNA transfection. First and foremost, the efficiency of siLDLR to downregulate *LDLR* in human LEC has been confirmed (Figure 6A). In total, siLDLR significantly upregulated 175 and downregulated 555 genes in human LEC (Figure 6B) with a fold change set at 1.5. Protein-protein interaction network (STRING database) based on RNA-transcriptomic analysis reveals that genes that are downregulated upon LDLR silencing are implicated in the cell cycle process, vesicle-mediated transport regulated exocytosis and cellular and metabolic processes (Figure 6D and Table 2). Among these genes, *VEGF-C* and *VEGFR-2* (*KDR*) were downregulated as well as *STAT3*. STAT3 binds to VEGF-C promoter region and modulate lymphangiogenesis 93. As VEGF-C binds to VEGFR-3 and lead to its regulation 94, the downregulation of *VEGF-C* by siLDLR could explain the downregulation of VEGFR-3 protein. These data suggest that the lack of VEGF-C lead to a downregulation of VEGFR-3 and VEGFR-2. Transcriptomic analysis also shows an upregulation of genes involved in cholesterol biosynthesis and cellular and metabolic processes (Figure 6C). Among the cholesterol regulating genes (*MSMO1, TM7SF2, HMGCS1, DHCR7, ACAT2, IDI1* and* FDFT1*, Figure 6E), *MSMO1* is one of most upregulated. Methylsterol monoxygenase 1 (MSMO1 previously SC4MOL) is localized in the endoplasmic reticulum (ER) membrane and is related to cholesterol biosynthesis I pathway by catalyzing the three-step monooxygenation required for the demethylation of 4,4-dimethyl and 4alpha-methylsterols, which can be subsequently metabolized to cholesterol 95. Hydroxymethylglutaryl-CoA synthase 1 (HMGCS1) is also implicated in cholesterol biosynthesis. *HMGCS1* in an enzyme which catalyses the production of mevalonate 96. It was recently shown that *HMGCS1* is a target to promote endothelial function in human umbilical vein endothelial cells (HUVECs) 97. Based on our databank, *FABP3* gene also appears upregulated following siLDLR treatment. Heart-type fatty-acid-binding protein 3 (FABP3) is a protein carrier transporting fatty acids and lipophilic substances from the cytoplasm to the nucleus mainly to maintain energy supply 98. FABP3 was also found to regulate intramuscular fat content and improve insulin sensitivity 99, 100. Being the response gene of peroxisome proliferator-activated receptor gamma (PPARy), FABP3 was demonstrated to have a direct impact on atherosclerosis, obesity and diabetes 101-103. In human coronary artery endothelial cells (HCAECs), FABP3 regulates the transcriptional activities of lysophosphoatidic acid (LPA) targeting PPARy 104. *FABP3* deficiency was also found to limit atherosclerosis development via inhibition of foam cell formation 101. Furthermore, it was recently demonstrated that FABP3 mediate membrane lipid saturation, altering fluidity and inducing ER stress in skeletal muscle with aging 105. The overexpression of *FABP3* in muscles altered membrane lipid composition, in turn deteriorating muscle mass and force 105. Given the prerequisite role of lymphatic muscle cells in lymphatic vessel contractility, this mechanism could explain the impairment in lymphatic contraction we observe *in vivo* when LDLR is downregulated. As none of the β-oxidation, triglyceride synthesis or hydrolysis genes were significantly modulated upon *LDLR* silencing, the upregulation of *ELOVL6* rather suggests that because of the lipid deprivation, siLDLR-treated LEC need more energy and will increase the rate of fatty acid synthesis to provide more energetic substrates for energy production through beta-oxidation. In addition, because of the observed decreased triglycerides content, another explanation may be a greater hydrolysis of triglycerides that will participate to the maintenance of adequate intracellular fatty acid supply for energy production 106.

## Conclusion

Efficient lymphatic drainage is crucial for atherosclerosis regression 11, 12, 107. Whereas hypercholesterolemia has previously been defined as the corner stone between cardiovascular disease and lymphatic function 10, 13, 14, it is now known that lymphatic function is rather tightly regulated throughout the whole development of the atherosclerosis process. We herein sought to determine whether and how lymphatic endothelial-LDLR might modulate lymphatic function. We are reporting that PCSK9 is needed to reduce lymphatic contraction capacity in endothelial-specific LDLR knockdown mice. *In vitro*, exogenous PCSK9 only reduced LEC-LDLR at the protein level and did not affect lymphatic endothelial cell integrity. This puts forward that PCSK9 may act upon lymphatic muscle cells to mediate its effect on lymphatic contraction capacity *in vivo*. Rather, knocking down the expression of *LDLR* mRNA in human LEC decreased VEGFR-3 expression and modulated cellular lipids. Lipidomic and transcriptomic analysis revealed changes in lipid rafts and genes that are involved in cholesterol biosynthesis and cellular and metabolic processes upon *LDLR* mRNA downregulation in LEC. Our results suggest that treatments that specifically palliate the downregulation of *Ldlr* mRNA by LEC preserve the integrity of the lymphatic endothelium and sustain lymphatic function in chronic inflammatory conditions such as atherosclerosis.

## Supplementary Material

Supplementary figures.

## Figures and Tables

**Table 1:**
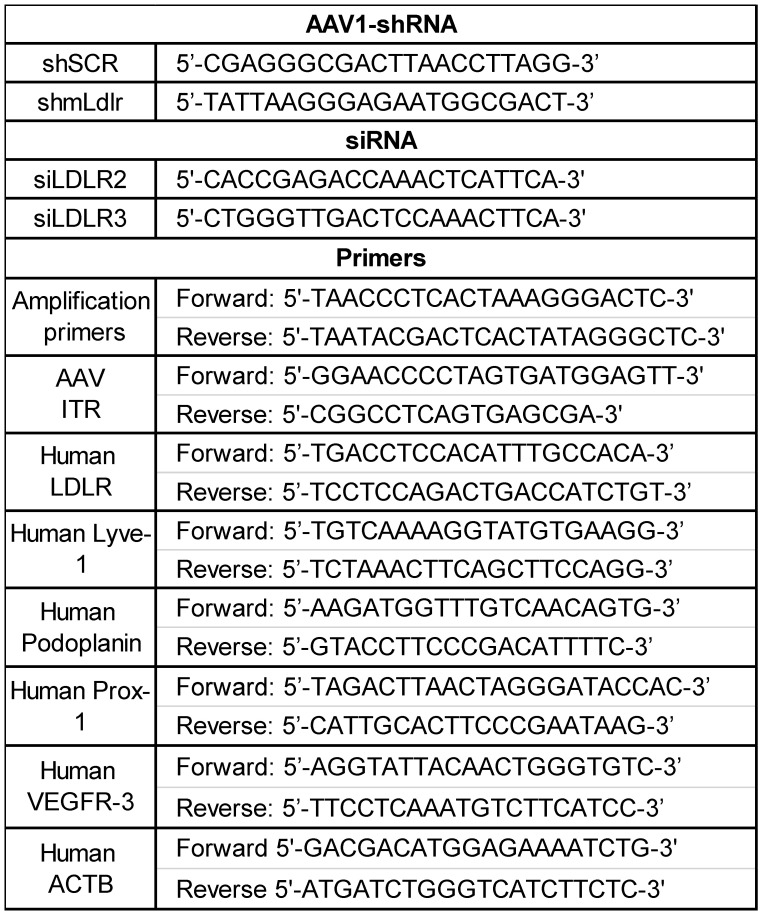
List of sequences for AAV1-shRNA, siRNA and primers used throughout the study. AAV, Adeno-associated virus

**Table 2:**
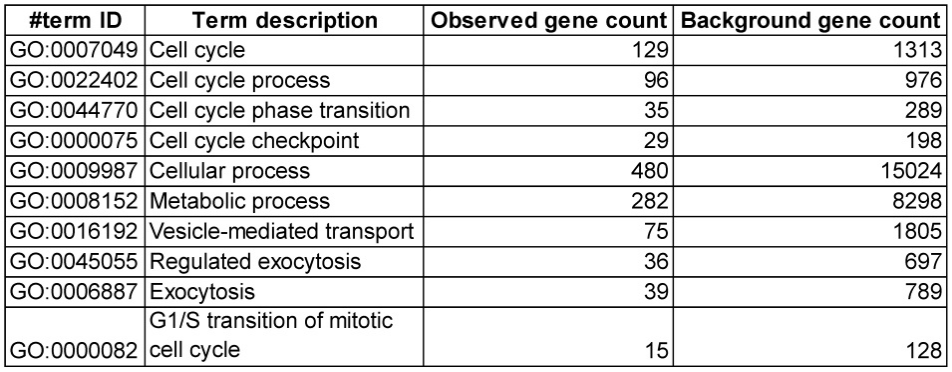
Main biological process that are downregulated by siLDLR treatment compared to siCtl. n = 3

## Abbreviations

AAV1: Adeno-Associated Virus type 1
CVD: cardiovascular disease
EV: extracellular vesicles
FBS: fetal bovine serum
FITC: fluorescein isothyiocyanate
HEK: human embryonic kidney
HuH7: hepatocytes
LDL: low-density lipoprotein
LDLR: low-density lipoprotein receptor
LEC: lymphatic endothelial cell
LN: lymph node
LVs: lymphatic vessels
LYVE-1: lymphatic vessel endothelial hyaluronic acid receptor-1
MFI: mean fluorescence intensity
PCSK9: proprotein convertase subtilisin kexin type 9
PDPN: podoplanin
ROI: region of interest
shRNA: short hairpin RNA
shSCR: sramble short hairpin RNA
siRNA: short-interfering RNA
VEGF-C 152S: vascular endothelial growth factor receptor-C
VEGFR-3: vascular endothelial growth factor receptor 3
Wt: C57BL/6 wild-type

## References

[1] Lemole GM (1981). The role of lymphstasis in atherogenesis. Ann Thorac Surg.

[2] Lemole GM Sr (2016). The Role of Lymphstasis in Atherogenesis Revisited. Ann Thorac Surg.

[3] Cueni LN, Detmar M (2008). The lymphatic system in health and disease. Lymphat Res Biol.

[4] Iqbal J, Hussain MM (2009). Intestinal lipid absorption. Am J Physiol Endocrinol Metab.

[5] Nakano T, Nakashima Y, Yonemitsu Y, Sumiyoshi S, Chen YX, Akishima Y (2005). Angiogenesis and lymphangiogenesis and expression of lymphangiogenic factors in the atherosclerotic intima of human coronary arteries. Hum Pathol.

[6] Eliska O, Eliskova M, Miller AJ (2006). The absence of lymphatics in normal and atherosclerotic coronary arteries in man: a morphologic study. Lymphology.

[7] Kholova I, Dragneva G, Cermakova P, Laidinen S, Kaskenpaa N, Hazes T (2011). Lymphatic vasculature is increased in heart valves, ischaemic and inflamed hearts and in cholesterol-rich and calcified atherosclerotic lesions. Eur J Clin Invest.

[8] Drozdz K, Janczak D, Dziegiel P, Podhorska M, Patrzalek D, Ziolkowski P (2008). Adventitial lymphatics of internal carotid artery in healthy and atherosclerotic vessels. Folia Histochem Cytobiol.

[9] Martel C, Li W, Fulp B, Platt AM, Gautier EL, Westerterp M (2013). Lymphatic vasculature mediates macrophage reverse cholesterol transport in mice. J Clin Invest.

[10] Lim HY, Thiam CH, Yeo KP, Bisoendial R, Hii CS, McGrath KC (2013). Lymphatic vessels are essential for the removal of cholesterol from peripheral tissues by SR-BI-mediated transport of HDL. Cell Metab.

[11] Martel C, Randolph GJ (2013). Atherosclerosis and transit of HDL through the lymphatic vasculature. Curr Atheroscler Rep.

[12] Yeo KP, Lim HY, Thiam CH, Azhar SH, Tan C, Tang Y (2020). Efficient aortic lymphatic drainage is necessary for atherosclerosis regression induced by ezetimibe. Sci Adv.

[13] Vuorio T, Nurmi H, Moulton K, Kurkipuro J, Robciuc MR, Ohman M (2014). Lymphatic vessel insufficiency in hypercholesterolemic mice alters lipoprotein levels and promotes atherogenesis. Arterioscler Thromb Vasc Biol.

[14] Lim HY, Rutkowski JM, Helft J, Reddy ST, Swartz MA, Randolph GJ (2009). Hypercholesterolemic mice exhibit lymphatic vessel dysfunction and degeneration. Am J Pathol.

[15] Milasan A, Smaani A, Martel C (2019). Early rescue of lymphatic function limits atherosclerosis progression in Ldlr(-/-) mice. Atherosclerosis.

[16] Milasan A, Dallaire F, Mayer G, Martel C (2016). Effects of LDL Receptor Modulation on Lymphatic Function. Sci Rep.

[17] Abifadel M, Varret M, Rabes JP, Allard D, Ouguerram K, Devillers M (2003). Mutations in PCSK9 cause autosomal dominant hypercholesterolemia. Nat Genet.

[18] Nassoury N, Blasiole DA, Tebon Oler A, Benjannet S, Hamelin J, Poupon V (2007). The cellular trafficking of the secretory proprotein convertase PCSK9 and its dependence on the LDLR. Traffic.

[19] Mayer G, Poirier S, Seidah NG (2008). Annexin A2 is a C-terminal PCSK9-binding protein that regulates endogenous low density lipoprotein receptor levels. J Biol Chem.

[20] Poirier S, Mamarbachi M, Chen WT, Lee AS, Mayer G (2015). GRP94 Regulates Circulating Cholesterol Levels through Blockade of PCSK9-Induced LDLR Degradation. Cell Rep.

[21] Demers A, Samami S, Lauzier B, Des Rosiers C, Ngo Sock ET, Ong H (2015). PCSK9 Induces CD36 Degradation and Affects Long-Chain Fatty Acid Uptake and Triglyceride Metabolism in Adipocytes and in Mouse Liver. Arterioscler Thromb Vasc Biol.

[22] Roubtsova A, Munkonda MN, Awan Z, Marcinkiewicz J, Chamberland A, Lazure C (2011). Circulating proprotein convertase subtilisin/kexin 9 (PCSK9) regulates VLDLR protein and triglyceride accumulation in visceral adipose tissue. Arterioscler Thromb Vasc Biol.

[23] Levy E, Ben Djoudi Ouadda A, Spahis S, Sane AT, Garofalo C, Grenier E (2013). PCSK9 plays a significant role in cholesterol homeostasis and lipid transport in intestinal epithelial cells. Atherosclerosis.

[24] Canuel M, Sun X, Asselin MC, Paramithiotis E, Prat A, Seidah NG (2013). Proprotein convertase subtilisin/kexin type 9 (PCSK9) can mediate degradation of the low density lipoprotein receptor-related protein 1 (LRP-1). PLoS One.

[25] Poirier S, Mayer G, Benjannet S, Bergeron E, Marcinkiewicz J, Nassoury N (2008). The proprotein convertase PCSK9 induces the degradation of low density lipoprotein receptor (LDLR) and its closest family members VLDLR and ApoER2. J Biol Chem.

[26] Lakoski SG, Lagace TA, Cohen JC, Horton JD, Hobbs HH (2009). Genetic and metabolic determinants of plasma PCSK9 levels. J Clin Endocrinol Metab.

[27] Baass A, Dubuc G, Tremblay M, Delvin EE, O'Loughlin J, Levy E (2009). Plasma PCSK9 is associated with age, sex, and multiple metabolic markers in a population-based sample of children and adolescents. Clin Chem.

[28] Chong C, Scholkmann F, Bachmann SB, Luciani P, Leroux JC, Detmar M (2016). *In vivo* visualization and quantification of collecting lymphatic vessel contractility using near-infrared imaging. Sci Rep.

[29] Riccardi C, Nicoletti I (2006). Analysis of apoptosis by propidium iodide staining and flow cytometry. Nat Protoc.

[30] Poncelet P, Robert S, Bailly N, Garnache-Ottou F, Bouriche T, Devalet B (2015). Tips and tricks for flow cytometry-based analysis and counting of microparticles. Transfus Apher Sci.

[31] Poncelet P, Robert S, Bouriche T, Bez J, Lacroix R, Dignat-George F (2016). Standardized counting of circulating platelet microparticles using currently available flow cytometers and scatter-based triggering: Forward or side scatter?. Cytometry A.

[32] Beuthan J, Minet O, Helfmann J, Herrig M, Muller G (1996). The spatial variation of the refractive index in biological cells. Phys Med Biol.

[33] Morales-Kastresana A, Telford B, Musich TA, McKinnon K, Clayborne C, Braig Z (2017). Labeling Extracellular Vesicles for Nanoscale Flow Cytometry. Sci Rep.

[34] Bolger AM, Lohse M, Usadel B (2014). Trimmomatic: a flexible trimmer for Illumina sequence data. Bioinformatics.

[35] Dobin A, Davis CA, Schlesinger F, Drenkow J, Zaleski C, Jha S (2013). STAR: ultrafast universal RNA-seq aligner. Bioinformatics.

[36] Li B, Dewey CN (2011). RSEM: accurate transcript quantification from RNA-Seq data with or without a reference genome. BMC Bioinformatics.

[37] Love MI, Huber W, Anders S (2014). Moderated estimation of fold change and dispersion for RNA-seq data with DESeq2. Genome Biol.

[38] Forest A, Ruiz M, Bouchard B, Boucher G, Gingras O, Daneault C (2018). Comprehensive and Reproducible Untargeted Lipidomic Workflow Using LC-QTOF Validated for Human Plasma Analysis. J Proteome Res.

[39] Solari E, Marcozzi C, Bartolini B, Viola M, Negrini D, Moriondo A (2020). Acute Exposure of Collecting Lymphatic Vessels to Low-Density Lipoproteins Increases Both Contraction Frequency and Lymph Flow: An *In vivo* Mechanical Insight. Lymphat Res Biol.

[40] Randolph GJ, Miller NE (2014). Lymphatic transport of high-density lipoproteins and chylomicrons. J Clin Invest.

[41] Roubtsova A, Chamberland A, Marcinkiewicz J, Essalmani R, Fazel A, Bergeron JJ (2015). PCSK9 deficiency unmasks a sex- and tissue-specific subcellular distribution of the LDL and VLDL receptors in mice. J Lipid Res.

[42] Milasan A, Ledoux J, Martel C (2015). Lymphatic network in atherosclerosis: the underestimated path. Future Sci OA.

[43] Ding Z, Liu S, Wang X, Deng X, Fan Y, Sun C (2015). Hemodynamic shear stress via ROS modulates PCSK9 expression in human vascular endothelial and smooth muscle cells and along the mouse aorta. Antioxid Redox Signal.

[44] Zadelaar S, Kleemann R, Verschuren L, de Vries-Van der Weij J, van der Hoorn J, Princen HM (2007). Mouse models for atherosclerosis and pharmaceutical modifiers. Arterioscler Thromb Vasc Biol.

[45] Gasheva OY, Zawieja DC, Gashev AA (2006). Contraction-initiated NO-dependent lymphatic relaxation: a self-regulatory mechanism in rat thoracic duct. J Physiol.

[46] Zawieja DC, Greiner ST, Davis KL, Hinds WM, Granger HJ (1991). Reactive oxygen metabolites inhibit spontaneous lymphatic contractions. Am J Physiol.

[47] Liao S, Cheng G, Conner DA, Huang Y, Kucherlapati RS, Munn LL (2011). Impaired lymphatic contraction associated with immunosuppression. Proc Natl Acad Sci U S A.

[48] Wang Y, Oliver G (2010). Current views on the function of the lymphatic vasculature in health and disease. Genes Dev.

[49] Morfoisse F, Tatin F, Chaput B, Therville N, Vaysse C, Metivier R (2018). Lymphatic Vasculature Requires Estrogen Receptor-alpha Signaling to Protect From Lymphedema. Arterioscler Thromb Vasc Biol.

[50] Li C, Briggs MR, Ahlborn TE, Kraemer FB, Liu J (2001). Requirement of Sp1 and estrogen receptor alpha interaction in 17beta-estradiol-mediated transcriptional activation of the low density lipoprotein receptor gene expression. Endocrinology.

[51] Kleinert H, Wallerath T, Euchenhofer C, Ihrig-Biedert I, Li H, Forstermann U (1998). Estrogens increase transcription of the human endothelial NO synthase gene: analysis of the transcription factors involved. Hypertension.

[52] Blenck CL, Harvey PA, Reckelhoff JF, Leinwand LA (2016). The Importance of Biological Sex and Estrogen in Rodent Models of Cardiovascular Health and Disease. Circ Res.

[53] Ferri N, Tibolla G, Pirillo A, Cipollone F, Mezzetti A, Pacia S (2012). Proprotein convertase subtilisin kexin type 9 (PCSK9) secreted by cultured smooth muscle cells reduces macrophages LDLR levels. Atherosclerosis.

[54] Schulz R, Schluter KD, Laufs U (2015). Molecular and cellular function of the proprotein convertase subtilisin/kexin type 9 (PCSK9). Basic Res Cardiol.

[55] Ding Z, Wang X, Liu S, Zhou S, Kore RA, Mu S (2020). NLRP3 inflammasome via IL-1beta regulates PCSK9 secretion. Theranostics.

[56] Giunzioni I, Tavori H, Covarrubias R, Major AS, Ding L, Zhang Y (2016). Local effects of human PCSK9 on the atherosclerotic lesion. J Pathol.

[57] Burke AC, Dron JS, Hegele RA, Huff MW (2017). PCSK9: Regulation and Target for Drug Development for Dyslipidemia. Annu Rev Pharmacol Toxicol.

[58] Bittner V (2016). Pleiotropic Effects of PCSK9 (Proprotein Convertase Subtilisin/Kexin Type 9) Inhibitors?. Circulation.

[59] Cheng JM, Oemrawsingh RM, Garcia-Garcia HM, Boersma E, van Geuns RJ, Serruys PW (2016). PCSK9 in relation to coronary plaque inflammation: Results of the ATHEROREMO-IVUS study. Atherosclerosis.

[60] Lambert G, Petrides F, Chatelais M, Blom DJ, Choque B, Tabet F (2014). Elevated plasma PCSK9 level is equally detrimental for patients with nonfamilial hypercholesterolemia and heterozygous familial hypercholesterolemia, irrespective of low-density lipoprotein receptor defects. J Am Coll Cardiol.

[61] Ferri N, Marchiano S, Tibolla G, Baetta R, Dhyani A, Ruscica M (2016). PCSK9 knock-out mice are protected from neointimal formation in response to perivascular carotid collar placement. Atherosclerosis.

[62] Munro S (2003). Lipid rafts: elusive or illusive?. Cell.

[63] Brown MS, Goldstein JL (1975). Regulation of the activity of the low density lipoprotein receptor in human fibroblasts. Cell.

[64] Shimomura I, Bashmakov Y, Shimano H, Horton JD, Goldstein JL, Brown MS (1997). Cholesterol feeding reduces nuclear forms of sterol regulatory element binding proteins in hamster liver. Proc Natl Acad Sci U S A.

[65] Hamada K, Oike Y, Takakura N, Ito Y, Jussila L, Dumont DJ (2000). VEGF-C signaling pathways through VEGFR-2 and VEGFR-3 in vasculoangiogenesis and hematopoiesis. Blood.

[66] Breslin JW, Gaudreault N, Watson KD, Reynoso R, Yuan SY, Wu MH (2007). Vascular endothelial growth factor-C stimulates the lymphatic pump by a VEGF receptor-3-dependent mechanism. Am J Physiol Heart Circ Physiol.

[67] Dieterich LC, Ducoli L, Shin JW, Detmar M (2017). Distinct transcriptional responses of lymphatic endothelial cells to VEGFR-3 and VEGFR-2 stimulation. Sci Data.

[68] Schuster S, Rubil S, Endres M, Princen HMG, Boeckel JN, Winter K (2019). Anti-PCSK9 antibodies inhibit pro-atherogenic mechanisms in APOE*3Leiden.CETP mice. Sci Rep.

[69] Safaeian L, Vaseghi G, Jabari H, Dana N (2019). Evolocumab, a proprotein convertase subtilisin/kexin type 9 inhibitor, promotes angiogenesis *in vitro*. Can J Physiol Pharmacol.

[70] Maxwell KN, Breslow JL (2004). Adenoviral-mediated expression of Pcsk9 in mice results in a low-density lipoprotein receptor knockout phenotype. Proc Natl Acad Sci U S A.

[71] Liu X, Li G, Ai L, Ye Q, Yu T, Yang B (2019). Prognostic value of ATAD3 gene cluster expression in hepatocellular carcinoma. Oncol Lett.

[72] Desai R, Frazier AE, Durigon R, Patel H, Jones AW, Dalla Rosa I (2017). ATAD3 gene cluster deletions cause cerebellar dysfunction associated with altered mitochondrial DNA and cholesterol metabolism. Brain.

[73] Brown MS, Goldstein JL (1974). Suppression of 3-hydroxy-3-methylglutaryl coenzyme A reductase activity and inhibition of growth of human fibroblasts by 7-ketocholesterol. J Biol Chem.

[74] Chen HW, Kandutsch AA, Waymouth C (1974). Inhibition of cell growth by oxygenated derivatives of cholesterol. Nature.

[75] Vitols S, Norgren S, Juliusson G, Tatidis L, Luthman H (1994). Multilevel regulation of low-density lipoprotein receptor and 3-hydroxy-3-methylglutaryl coenzyme A reductase gene expression in normal and leukemic cells. Blood.

[76] Martinez-Botas J, Suarez Y, Ferruelo AJ, Gomez-Coronado D, Lasuncion MA (1999). Cholesterol starvation decreases p34(cdc2) kinase activity and arrests the cell cycle at G2. FASEB J.

[77] Corsini A, Mazzotti M, Raiteri M, Soma MR, Gabbiani G, Fumagalli R (1993). Relationship between mevalonate pathway and arterial myocyte proliferation: *in vitro* studies with inhibitors of HMG-CoA reductase. Atherosclerosis.

[78] Jakobisiak M, Bruno S, Skierski JS, Darzynkiewicz Z (1991). Cell cycle-specific effects of lovastatin. Proc Natl Acad Sci U S A.

[79] Chakrabarti R, Engleman EG (1991). Interrelationships between mevalonate metabolism and the mitogenic signaling pathway in T lymphocyte proliferation. J Biol Chem.

[80] Luan C, Chen X, Zhu Y, Osland JM, Gerber SD, Dodds M (2019). Potentiation of Psoriasis-Like Inflammation by PCSK9. J Invest Dermatol.

[81] Guo Y, Tang Z, Yan B, Yin H, Tai S, Peng J PCSK9 (Proprotein Convertase Subtilisin/Kexin Type 9) Triggers Vascular Smooth Muscle Cell Senescence and Apoptosis: Implication of Its Direct Role in Degenerative Vascular Disease. Arterioscler Thromb Vasc Biol. 2021: Atvbaha121316902.

[82] Karaman S, Leppanen VM, Alitalo K (2018). Vascular endothelial growth factor signaling in development and disease. Development.

[83] Zabroski IO, Nugent MA (2021). Lipid Raft Association Stabilizes VEGF Receptor 2 in Endothelial Cells. Int J Mol Sci.

[84] Pralle A, Keller P, Florin EL, Simons K, Horber JK (2000). Sphingolipid-cholesterol rafts diffuse as small entities in the plasma membrane of mammalian cells. J Cell Biol.

[85] Sorci-Thomas MG, Thomas MJ (2016). Microdomains, Inflammation, and Atherosclerosis. Circ Res.

[86] van der Veen JN, Kennelly JP, Wan S, Vance JE, Vance DE, Jacobs RL (2017). The critical role of phosphatidylcholine and phosphatidylethanolamine metabolism in health and disease. Biochim Biophys Acta Biomembr.

[87] Miao JY, Kaji K, Hayashi H, Araki S (1997). Suppression of apoptosis by inhibition of phosphatidylcholine-specific phospholipase C in vascular endothelial cells. Endothelium.

[88] Li Z, Agellon LB, Allen TM, Umeda M, Jewell L, Mason A (2006). The ratio of phosphatidylcholine to phosphatidylethanolamine influences membrane integrity and steatohepatitis. Cell Metab.

[89] Cui Z, Houweling M, Chen MH, Record M, Chap H, Vance DE (1996). A genetic defect in phosphatidylcholine biosynthesis triggers apoptosis in Chinese hamster ovary cells. J Biol Chem.

[90] Simons K, Toomre D (2000). Lipid rafts and signal transduction. Nat Rev Mol Cell Biol.

[91] Hannun YA, Obeid LM (1995). Ceramide: an intracellular signal for apoptosis. Trends Biochem Sci.

[92] Obeid LM, Linardic CM, Karolak LA, Hannun YA (1993). Programmed cell death induced by ceramide. Science.

[93] Huang YH, Yang HY, Huang SW, Ou G, Hsu YF, Hsu MJ (2016). Interleukin-6 Induces Vascular Endothelial Growth Factor-C Expression via Src-FAK-STAT3 Signaling in Lymphatic Endothelial Cells. PLoS One.

[94] Joukov V, Pajusola K, Kaipainen A, Chilov D, Lahtinen I, Kukk E (1996). A novel vascular endothelial growth factor, VEGF-C, is a ligand for the Flt4 (VEGFR-3) and KDR (VEGFR-2) receptor tyrosine kinases. Embo j.

[95] He M, Kratz LE, Michel JJ, Vallejo AN, Ferris L, Kelley RI (2011). Mutations in the human SC4MOL gene encoding a methyl sterol oxidase cause psoriasiform dermatitis, microcephaly, and developmental delay. J Clin Invest.

[96] Mathews ES, Mawdsley DJ, Walker M, Hines JH, Pozzoli M, Appel B (2014). Mutation of 3-hydroxy-3-methylglutaryl CoA synthase I reveals requirements for isoprenoid and cholesterol synthesis in oligodendrocyte migration arrest, axon wrapping, and myelin gene expression. J Neurosci.

[97] Ying X, Zhu Y, Jin X, Chang X (2021). Umbilical cord plasma-derived exosomes from preeclamptic women induce vascular dysfunction by targeting HMGCS1 in endothelial cells. Placenta.

[98] Shearer J, Fueger PT, Rottman JN, Bracy DP, Binas B, Wasserman DH (2005). Heart-type fatty acid-binding protein reciprocally regulates glucose and fatty acid utilization during exercise. Am J Physiol Endocrinol Metab.

[99] Li B, Zerby HN, Lee K (2007). Heart fatty acid binding protein is upregulated during porcine adipocyte development. J Anim Sci.

[100] Shearer J, Fueger PT, Bracy DP, Wasserman DH, Rottman JN (2005). Partial gene deletion of heart-type fatty acid-binding protein limits the severity of dietary-induced insulin resistance. Diabetes.

[101] Tan L, Lu J, Liu L, Li L Fatty acid binding protein 3 deficiency limits atherosclerosis development via macrophage foam cell formation inhibition. Experimental Cell Research. 2021: 112768.

[102] Zhang Y, Kent JW 2nd, Lee A, Cerjak D, Ali O, Diasio R (2013). Fatty acid binding protein 3 (fabp3) is associated with insulin, lipids and cardiovascular phenotypes of the metabolic syndrome through epigenetic modifications in a Northern European family population. BMC Med Genomics.

[103] Ozawa S, Ueda S, Li Y, Mori K, Asanuma K, Yanagita M (2014). Fatty acid binding protein 3 as a potential mediator for diabetic nephropathy in eNOS deficient mouse. Biochem Biophys Res Commun.

[104] Tsukahara R, Haniu H, Matsuda Y, Tsukahara T (2014). Heart-type fatty-acid-binding protein (FABP3) is a lysophosphatidic acid-binding protein in human coronary artery endothelial cells. FEBS Open Bio.

[105] Lee S-M, Lee SH, Jung Y, Lee Y, Yoon JH, Choi JY (2020). FABP3-mediated membrane lipid saturation alters fluidity and induces ER stress in skeletal muscle with aging. Nature Communications.

[106] Wong BW, Wang X, Zecchin A, Thienpont B, Cornelissen I, Kalucka J (2017). The role of fatty acid β-oxidation in lymphangiogenesis. Nature.

[107] Martel C, Li W, Fulp B, Platt AM, Gautier EL, Westerterp M (2013). Lymphatic vasculature mediates macrophage reverse cholesterol transport in mice. J Clin Invest.

