# Supplementary material for: Downregulation of low-density lipoprotein receptor mRNA in lymphatic endothelial cells impairs lymphatic function through changes in intracellular lipids
Source: Theranostics. 2022 Jan 1;12(3):1440–58. doi: 10.7150/thno.58780 (PMC8771568; doi:10.7150/thno.58780)
Supplement: Supplementary file 1 — Supplementary figures. [file thnov12p1440s1.pdf]

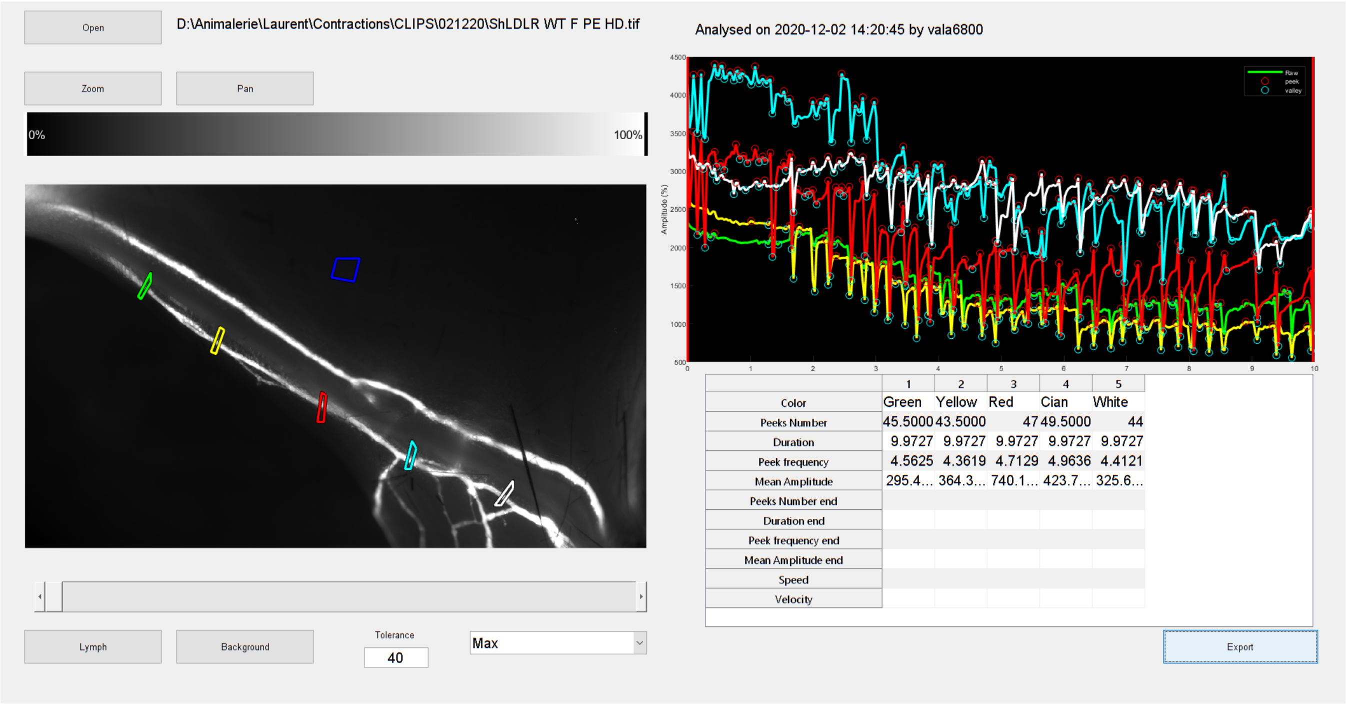

Figure S1. Representative screenshot of the LymphPulse 3.0 Matlab™ based software.

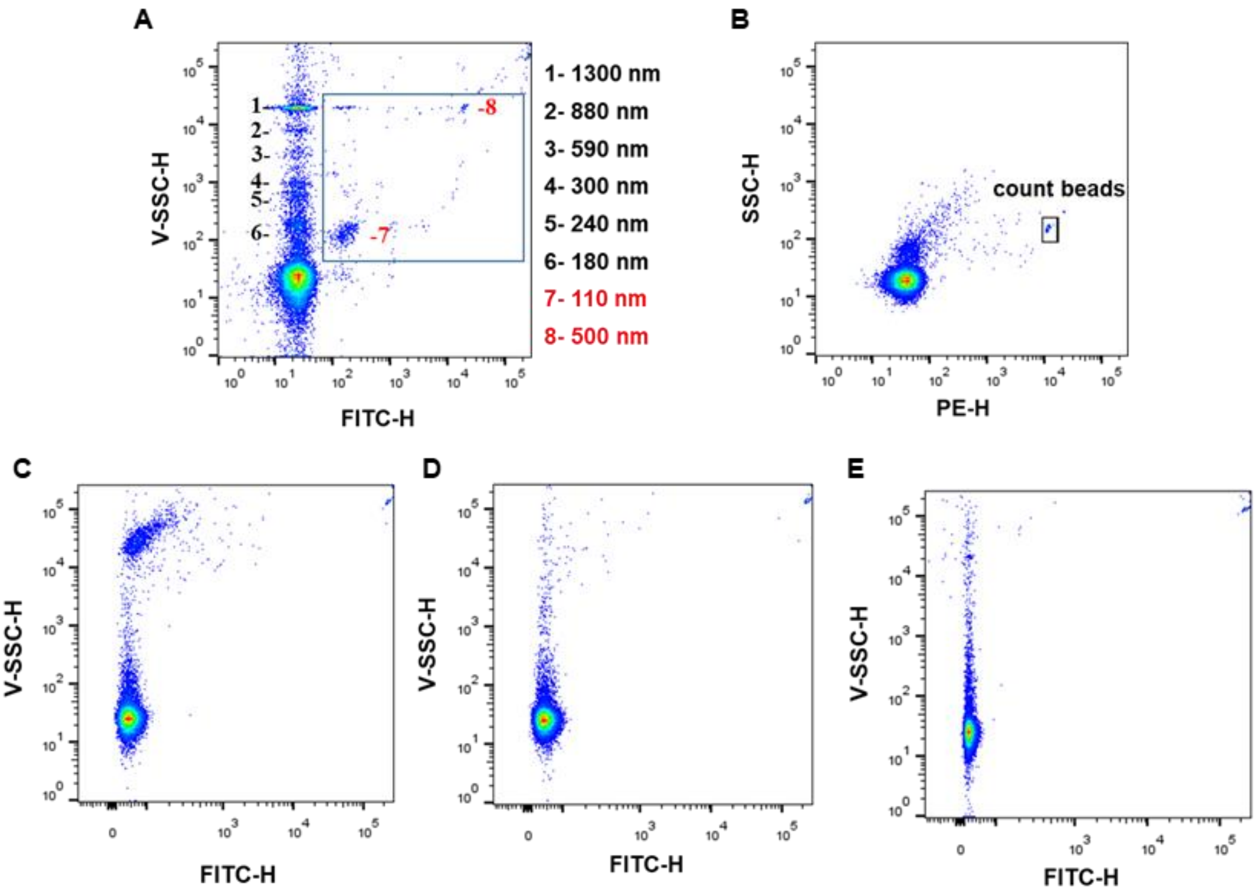

Figure S2. Cytometer setup for the measurement of extracellular vesicles in cell culture media. (A) The flow cytometer was calibrated for EV detection using the ApogeeMix containing non-fluorescent silica beads (black; 180, 240, 300, 590, 880, and 1300 nm) and FITC-fluorescent latex beads (red; 110 and 500 nm). **(B)** Fluorescent count beads (1 µM in diameter) were used to quantify the concentration of beads per sample. **(C)** Representative sample and **(D)** its Triton X-100 control. **(E)** Background control of antibody mix.

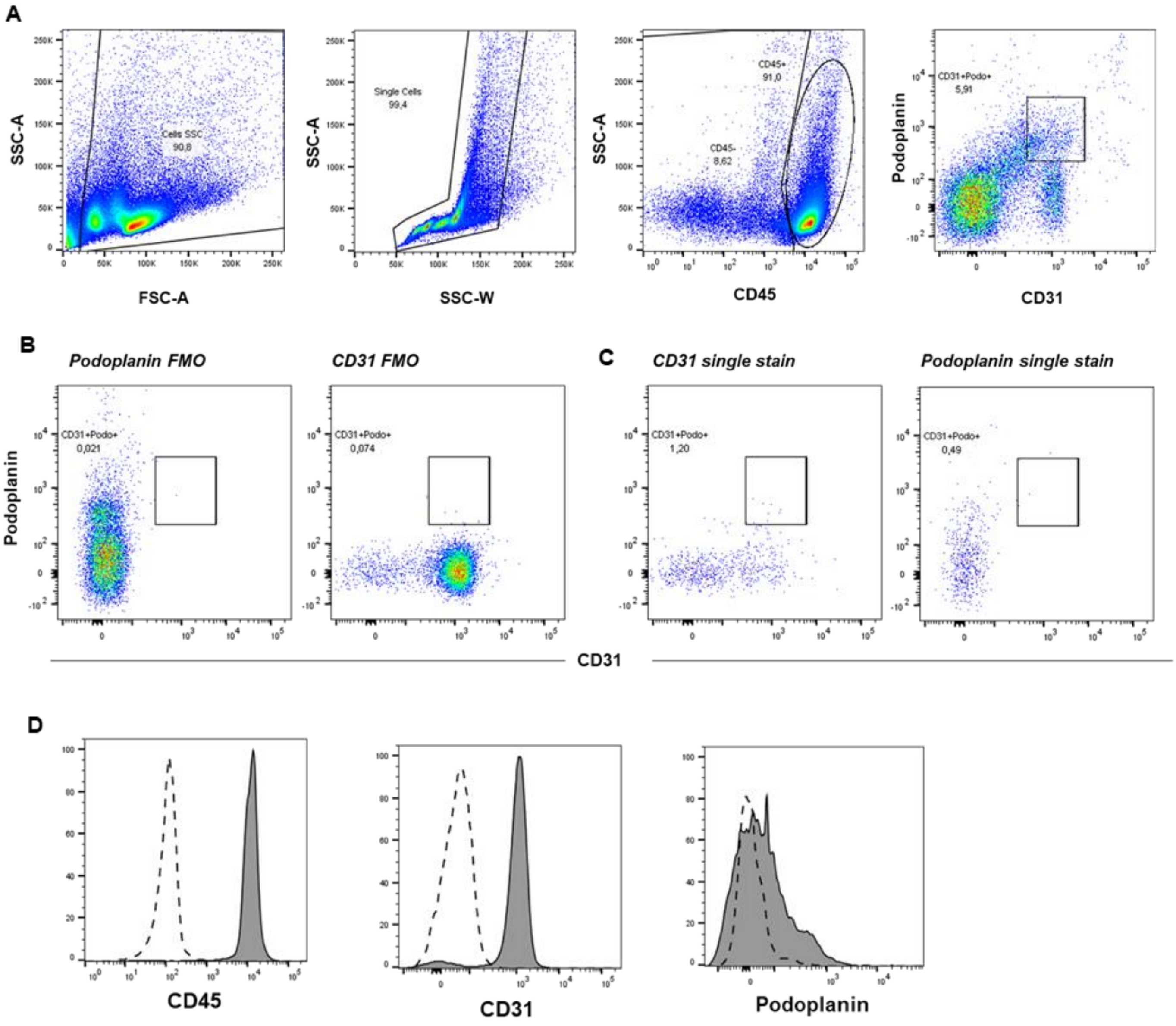

Figure S3. Assessement of membrane LDLR expression by flow cytometry. (A) Backgating strategy for the detection of membrane LDLR on lymphatic endothelial cells. **(B)** Fluorescence minus one (FMO) and **(C)** single stain controls were used to set the gates for the $CD31^{+}Podoplanin^{+}$ cells. **(D)** Representative histograms for CD45, CD31 and podoplanin FMO controls (dotted lines). n = 3.

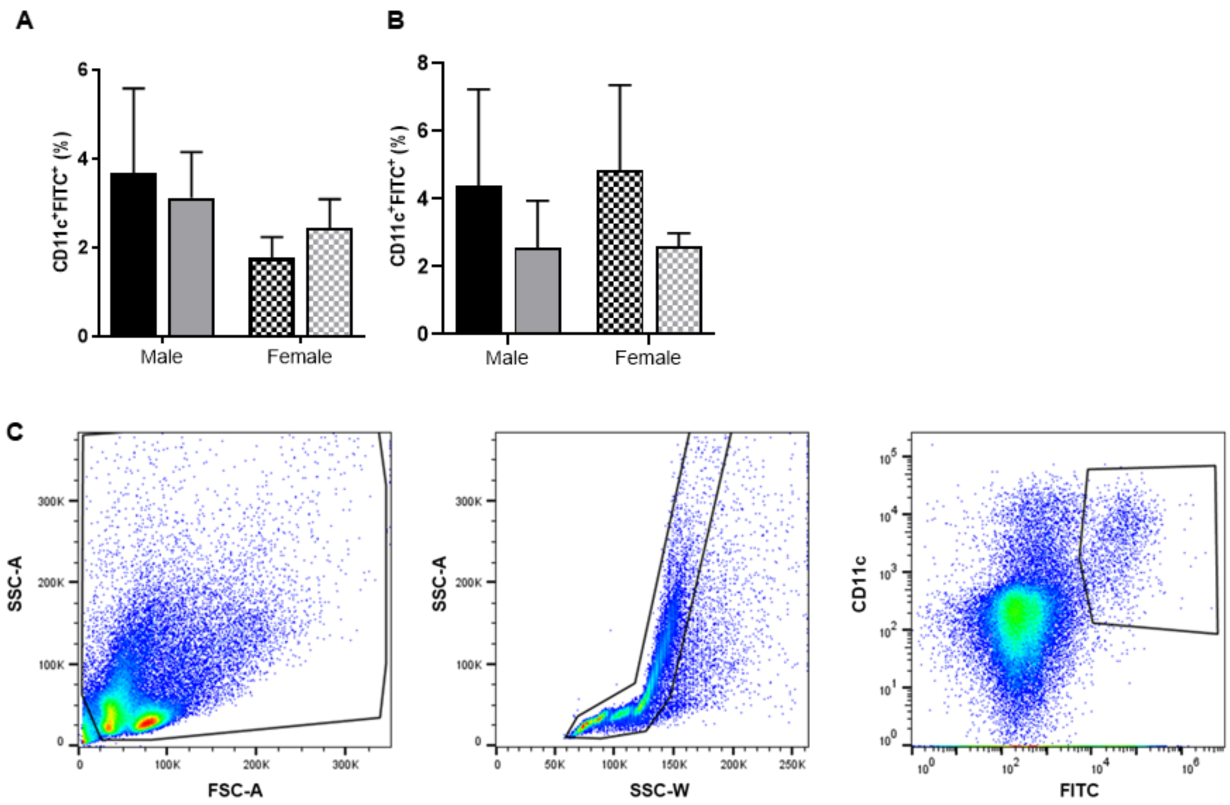

Figure S4. ***In vivo* assessment of dendritic cell transport in the lymphatics.** Two weeks after the intraperitoneal injection of an AAV1 containing a shLdlr or shSCR, dendritic cell migration was determined following a 18 h contact sensitization assay in **(A)** wild-type and **(B)** *Pcsk9*$^{-/-}$ mice. Black histogram, ShSCR and grey histogram, ShLdlr. **(C)** Representative dot plots of FITC^{+}CD11c^{+} cells in skin-draining LNs. n = 4-9.

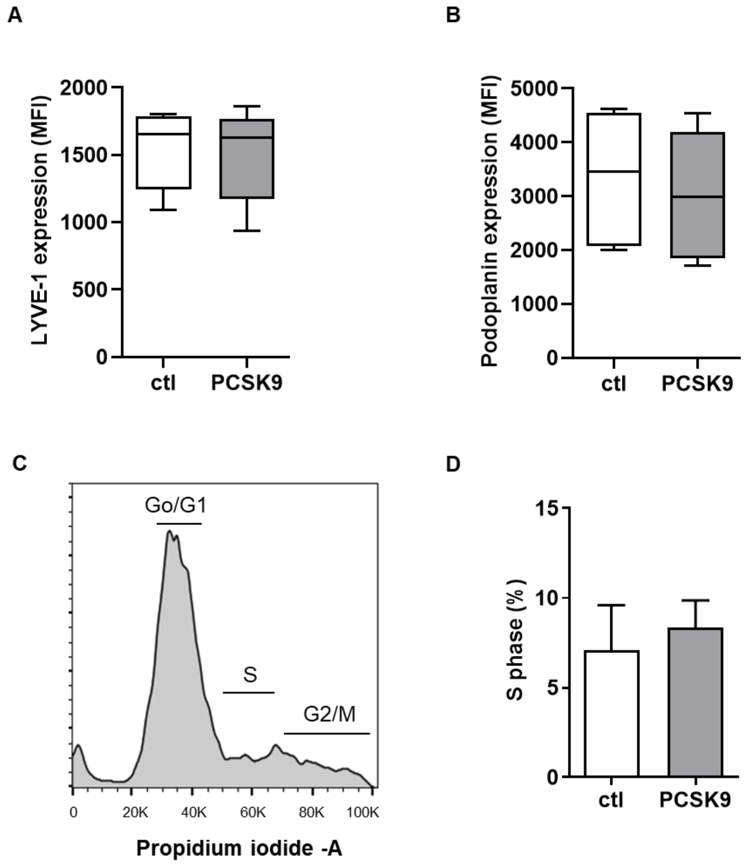

Figure S5. Effect of PCSK9 on human LEC markers and cell cycle distribution. Protein expression of **(A)** LYVE-1 and **(B)** podoplanin was measured in human LEC following a 16 h incubation with 6.5 µg/ml human recombinant PCSK9. **(C)** Representative histogram of the assessment of cell cycle performed by flow cytometry, and **(D)** quantification of the proportion of cells in the S phase following exogenous PCSK9 treatment. n = 3-7.
